# Spermidine stimulates hypocrellin A biosynthesis through nitric oxide signaling in *Shiraia* sp. S9

**DOI:** 10.1186/s40643-026-01051-2

**Published:** 2026-04-11

**Authors:** Li Ping Zheng, Rui Peng Cong, Xin Ping Li, Jian Qin Zhou, Jian Wen Wang

**Affiliations:** 1 Department of Horticultural Sciences, Soochow University, Suzhou, 215123 China; 2 College of Pharmaceutical Sciences, Soochow University, Suzhou, 215123 China; 3 College of Pharmaceutical Sciences, Dali University, Dali, 671000 China

**Keywords:** *Shiraia*, Hypocrellin A, Polyamines, Spermidine, Nitric oxide, Cyclic guanosine monophosphate

## Abstract

Hypocrellin A (HA), a photoactive perylenequinone from the bambusicolous *Shiraia* fungi*,* possesses potent photodynamic anticancer and antimicrobial properties. However, the signaling mechanisms governing its biosynthesis remain poorly understood. In this study, we identify spermidine (Spd), a ubiquitous polyamine, as a novel elicitor that significantly enhances HA production in *Shiraia* sp. S9. Spd activated both nitric oxide synthase (NOS) and nitrate reductase (NR) for nitric oxide (NO) generation, leading to the stimulation of the soluble guanylate cyclase (sGC)–cyclic guanosine monophosphate (cGMP) signaling cascade. Inhibition of NO generation or sGC activity suppressed both cGMP accumulation and HA biosynthesis. Transcriptomic analysis revealed that Spd-induced NO signaling upregulated genes in central carbon metabolism and the hypocrellin biosynthetic gene cluster. The dual elicitation strategy by the combined addition of Spd and the NO donor sodium nitroprusside (SNP) exhibited a strong enhancing effect, increasing HA yield by 4.6-fold compared with control cultures. These results demonstrate that Spd regulates HA biosynthesis through a NO–cGMP–mediated signaling pathway, unveiling polyamines as new metabolic elicitors and providing an efficient dual-elicitation strategy for large-scale hypocrellin production.

**Graphical abstract:**

**Supplementary Information:**

The online version contains supplementary material available at 10.1186/s40643-026-01051-2.

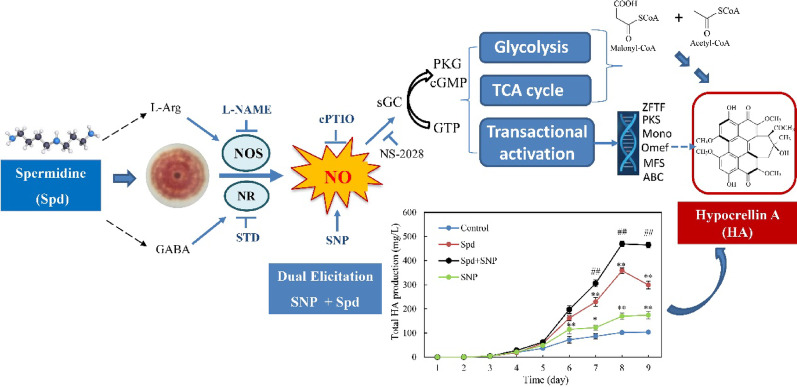

## Introduction

*Shiraia* fruiting bodies are rich in perylenequinone pigments including hypocrellins, elsinochromes and shiraiachromes, which are natural photosensitizers characterized by an oxidized pentacyclic core (Daub et al. 2013). Hypocrellins (HA-HD) and shiraiachrome A have been isolated from the fruiting bodies of the bambusicolous fungus *S. bambusicola* and *Hypocrella bambusae*, as well as other *Shiraia* endophytes (Wu et al. 1989; Li et al. 2025). These fruiting bodies, known as “Zhu Huang” in China, have been utilized for centuries in traditionally Chinese medicine to treat gastric ailments, blood stasis and rheumatoid arthritis (Tong et al. 2004). As non-porphyrin photosensitizers, hypocrellins exhibit exceptional photoactivated properties, including high quantum yields of reactive oxygen species (ROS) such as singlet oxygen (^1^O₂) and superoxide radical (O_2_^‧−^), coupled with low dark toxicity and rapid clearance from healthy tissues (Diwu and Lown 1990). HA demonstrates an excellent light-induced antimicrobial ability against azole-resistant *Candida albicans* and methicillin-resistant *Staphylococcus aureus* (Du et al. 2012). It has even been shown to inactivate human immunodeficiency virus (HIV), vesicular stomatitis virus and the severe acute respiratory syndrome coronavirus 2 (SARS-CoV-2) (Li et al. 2021). Both HA and HB inhibit various tumor cells, including hepatocellular carcinoma, human malignant epithelioid and lung adenocarcinoma cells, by inducing ROS-mediated apoptosis (Zhang et al. 2024). Furthermore, chemical modification and nano-formulations (e.g., liposomes, polymers, and micelles) have enhanced the solubility and delivery of these compounds, improving the efficacy of photodynamic therapy (PDT) on melanoma (Nkosi et al. 2024). Currently, hypocrellin ointments are used clinically as prescription PDT drugs in China for treating lichen amyloidosis and vulva lesions (Guan 2021). Beyond medicine, hypocrellins have extensive applications as photosensitive pesticides in agricultural and as natural colorants or preservatives in the food industries (Deng et al. 2022). The expanding range of biomedical applications has led to a rising demands for hypocrellin resources. However, production remains constrained by the difficulties of artificial cultivation and the complex of chemical synthesis (Mulrooney et al. 2012), leaving extraction from limited wild *Shiraia* fruiting bodies as the primary resource. Consequently, *Shiraia* mycelium cultures are being developed as a biotechnological alternative for hypocrellin production (Li et al. 2025). Although the solid-state and submerged liquid culture methods have been established and optimized for parameters such as the inoculum level, nutrient sources, pH value, temperature and incubation time, hypocrellin yields remain relatively lower (approximately 2–5 mg/g dry weight in solid-state fermentation or 10–40 mg/L in liquid fermentation (Liang et al. 2009; Yang et al. 2009; Cai et al. 2010). Some *Shiraia* isolates (*Shiraia* sp. SUPERH168 and *S. bambusicola* S8) even fail to produce hypocrellins entirely in submerged cultures (Cai et al. 2011; Lei et al. 2017). To address this bottleneck, various strategies including light (Sun et al. 2018; Wang et al. 2024) or ultrasound exposure (Sun et al. 2017), treatments of lanthanum chloride (Lu et al. 2019), Triton X-100 (Lei et al. 2017), bamboo charcoal powder (Li et al. 2019), and bacterial elicitation (Ma et al. 2019b; Zhou et al. 2023; Li et al. 2024), have been employed to induce hypocrellin production in mycelium cultures. Recent studies reported that exogenous L-arginine could significantly improve perylenequinone production (Chen et al. 2022). Since L-arginine is a major precursor for polyamine biosynthesis (Liu et al. 2015), we speculate that polyamines may regulate HA biosynthesis*.*

Polyamines are ubiquitous nitrogenous polycationic compounds, such as putrescine (Put), spermidine (Spd), and spermine (Spm), which are essential for cell growth and viability across eukaryotes (Igarashi and Kashiwagi 2015). In plants, polyamines act as biostimulants that regulate development and stress tolerance (Mustafavi et al. 2018; Liu et al. 2025). However, there was less report about their effects on fungal growth and secondary metabolism. Polyamines have been shown to influence spore germination (Rajam and Galston 1985), hyphal development (El Ghachtouli et al. 1996; Jiménez-Bremont et al. 2001), and the production of metabolites like penicillin (Martín et al. 2011), aflatoxin (Majumdar et al. 2012; Rajasekaran and Cary 2018), deoxynivalenol (Gardiner et al. 2009) in certain species. Recently, it was shown that the exogenous addition of Spd or 1,3-diaminopropane at 5 mM in mycelium cultures of *Acremonium chrysogenum* HY strain upregulated gene expressions in the β-lactam biosynthetic cluster and increased the production of cephalosporin C by 15–20% (Zhgun and Eldarov 2021. These results highlight the potential to harness polyamines for biotechnological applications in enhanced production of fungal bioactive compounds.

In *Shiraia* mycelium cultures*,* HA production was induced by L-valine (Shen et al. 2023), urea (Tang et al. 2024) and L-arginine (Chen et al. 2022), all of which are linked to polyamine biosynthesis, highlighting the potential regulatory role of polyamines*.* As a follow-up to our efforts to elucidate the signals for eliciting hypocrellin production (Li et al. 2025), we examined the effects of exogenous Put, Spd and Spm on *Shiraia* sp. S9. We optimized Spd treatment conditions and utilized comparative transcriptome sequencing to reveal the mechanism by which Spd improves HA production via nitric oxide (NO) signaling. Additionally, we established a combined elicitation strategy using Spd and the NO donor sodium nitroprusside (SNP) as a novel approach for enhancing HA yields in mycelium cultures.

## Materials and methods

### Strains and culture conditions

The fungal isolate *Shiraia* sp. S9 was obtained from freshly collected *Shiraia* fruiting bodies growing on bamboo (*Brachystachyum densiflorum*) at Tianmu mountain, Zhejiang Province, China, as previously described (Ma et al. 2019a). The strain has been deposited in the China General Microbiological Culture Collection Center (CGMCC) under accession number CGMCC 16369. Stock cultures were routinely maintained on potato dextrose agar (PDA) at 4 °C. For solid-state cultivation, a 5-mm mycelial disc excised from actively growing colonies was placed onto fresh PDA plates and incubated in the dark at 28 °C for 8 days. For submerged fermentation, 10% (v/v) of the seed culture was inoculated into 150-mL Erlenmeyer flasks containing 50 mL of production medium and cultured at 28 °C with agitation at 150 rpm. The preparation of seed cultures and the composition of the media were conducted according to previously established procedures (Sun et al. 2017).

### Polyamine treatments

Exogenous polyamines (Put, Spd and Spm) were sourced from Yuanye Bio-Technology Co. Ltd (Shanghai, China). For solid-state experiments, *Shiraia* sp. S9 was grown on PDA supplemented with 1.0 mM of each polyamine at 28 °C for 8 days. In liquid cultures, the timing of Spd addition was evaluated by treating mycelia with 1.0 mM Spd at various intervals from day 2 to day 7. To determine the optimal dosage, Spd concentrations (0.5–4.0 mM) were added to 4-day-old cultures. HA production was quantified on day 8.

### Determination of NO generation

Intracellular NO production in *Shiraia* sp. S9 hyphae was monitored using the NO-sensitive fluorescent dye 4,5-diaminofluorescein diacetate (DAF-2 DA; Sigma-Aldrich, St. Louis, USA). Freshly collected mycelia were rinsed with sterile distilled water and subsequently incubated with 10 μM DAF-2 DA in the dark for 30 min, following the method of Turrion-Gomez and Benito (2011). Fluorescence signals were observed and recorded using an Olympus CKX41 fluorescence microscope (Tokyo, Japan) with excitation at 480 nm and emission at 515 nm. Total NO levels were further quantified using a commercial Total Nitric Oxide Assay Kit (Beyotime Biotechnology, Nanjing, China) in accordance with the manufacturer’s instructions. To verify NO production and explore its potential signaling mechanisms, pharmacological modulators were applied 30 min before Spd treatment. These included the NO donor SNP (5 μM), the NO scavenger cPTIO (2-(4-carboxyphenyl)-4,4,5,5-tetramethylimidazoline-1-oxyl-3-oxide; 100 μM), the nitric oxide synthase (NOS) inhibitor L-NAME (100 μM), the nitrate reductase (NR) inhibitor sodium tungstate dihydrate (STD, 100 μM), the soluble guanylate cyclase (sGC) inhibitor NS-2028 (10 μM) and cGMP (5 mM). For enzymatic assays, mycelia were ground in liquid nitrogen and the activities of NOS and NR were determined using corresponding assay kits (Nanjing Jiancheng Institute of Bioengineering, Nanjing, China). Total protein concentrations were measured with an Enhanced BCA Protein Assay Kit (Beyotime Biotechnology, Shanghai, China) to normalize enzyme activities.

### Quantification of cyclic guanosine monophosphate (cGMP) and Spd metabolites

Intracellular cGMP content was determined using an ELISA Kit (Jiangsu Meimian Industrial Co., Ltd., Yancheng, China). For analysis on Spd-related metabolites: L‑arginine (L-Arg), glutamine (Gln) and γ-aminobutyric acid (GABA), frozen mycelia (0.1 g) were extracted with 0.5 mL of 75% ethanol at boiling temperature and the supernatant was collected after centrifugation for 10 min at 12,000 rpm. The sample (0.5 mL) was mixed with 1 mL sodium bicarbonate solution (0.5 M) and 1 mL 2,4-dinitrofluorobenzene (DNBF, 1%) in acetonitrile. The mixture was incubated at 60 °C for 1 h in darkness. After derivatization, 7.5 mL of phosphate buffer (0.1 M, pH 7.0) was added and analyzed by a reversed-phase HPLC method (Zhou et al. 2011).

### Transcriptome sequencing and analysis

RNA-seq was performed on hyphae after 3 days of treatment with 1.0 mM Spd or a combination of 1.0 mM Spd and 100 μM cPTIO. Three biological replicates for control, Spd treatment and Spd + cPTIO group were sampled. Complementary DNA (cDNA) libraries were prepared and sequenced using the Illumina HiSeq™ 2500 platform (Illumina, San Diego, USA). The raw sequencing datasets have been deposited in the National Center for Biotechnology Information (NCBI) under BioProject accession number PRJNA1065594. Transcriptome assembly was conducted de novo with the Trinity software package (version trinityrnaseq_r20131110) following the approach described by Grabherr et al. (2011). The resulting unigenes were annotated by comparison against public databases, including the Kyoto Encyclopedia of Genes and Genomes (KEGG) and Gene Ontology (GO), using BLASTX with an E-value threshold of ≤ 1 × 10⁻^5^ (Altschul et al. 1990; Kanehisa et al. 2008). Gene expression levels were estimated based on both raw read counts and fragments per kilobase of transcript per million mapped reads (FPKM). Differentially expressed genes (DEGs) were identified according to the criteria of |log₂(fold change)|≥ 1 and an adjusted *p*-value ≤ 0.05, as described by Roberts and Pachter (2013).

### Extraction and quantification of perylenequinones

Perylenequinones were extracted from PDA plates, hyphae and cultural broth according to method described by Chen et al. (2022). The individual perylenequinone was analyzed by a reverse-phase Agilent 1260 HPLC system (Agilent Co., Wilmington, USA) equipped with the Agilent HC-C18 column (250 × 4.6 mm dimension) at 465 nm according to the procedure described by Tong et al. (2017). HA-HC standards were obtained from the Chinese National Compound Library (purity > 98%, Shanghai, China). Elsinochrome A-C (EA-EC; 98%) were purchased from Angene International Limited (London, UK). Total HA production was calculated as the sum of intracellular and extracellular concentrations.

### Quantitative real‑time PCR (qRT‑PCR)

Total RNA was extracted using the RNAprep pure Plant Kit (Tiangen, Beijing, China) and reverse transcribed into cDNA using a ReverTraAce qPCR RT Kit (Toyobo, Osaka, Japan). qRT-PCR was conducted on a CFX96 Touch Real-Time PCR Detection System (Bio-Rad, USA) (Ma et al. 2018). Primer sequences used for qRT-PCR are listed in Table S1. Expression levels were calculated using the 2^−△△CT^ method (Livak and Schmittgen 2001).

### Statistical analysis

All experiments were conducted with three independent biological replicates, each consisting of ten plates or flasks. Data are presented as the mean ± standard deviation (SD). Statistical analyses were performed using Student’s *t*-test, and differences were considered statistically significant at *p* < 0.05.

## Results

### Effects of polyamines on *Shiraia* perylenequinone production

**Fig. 1:**
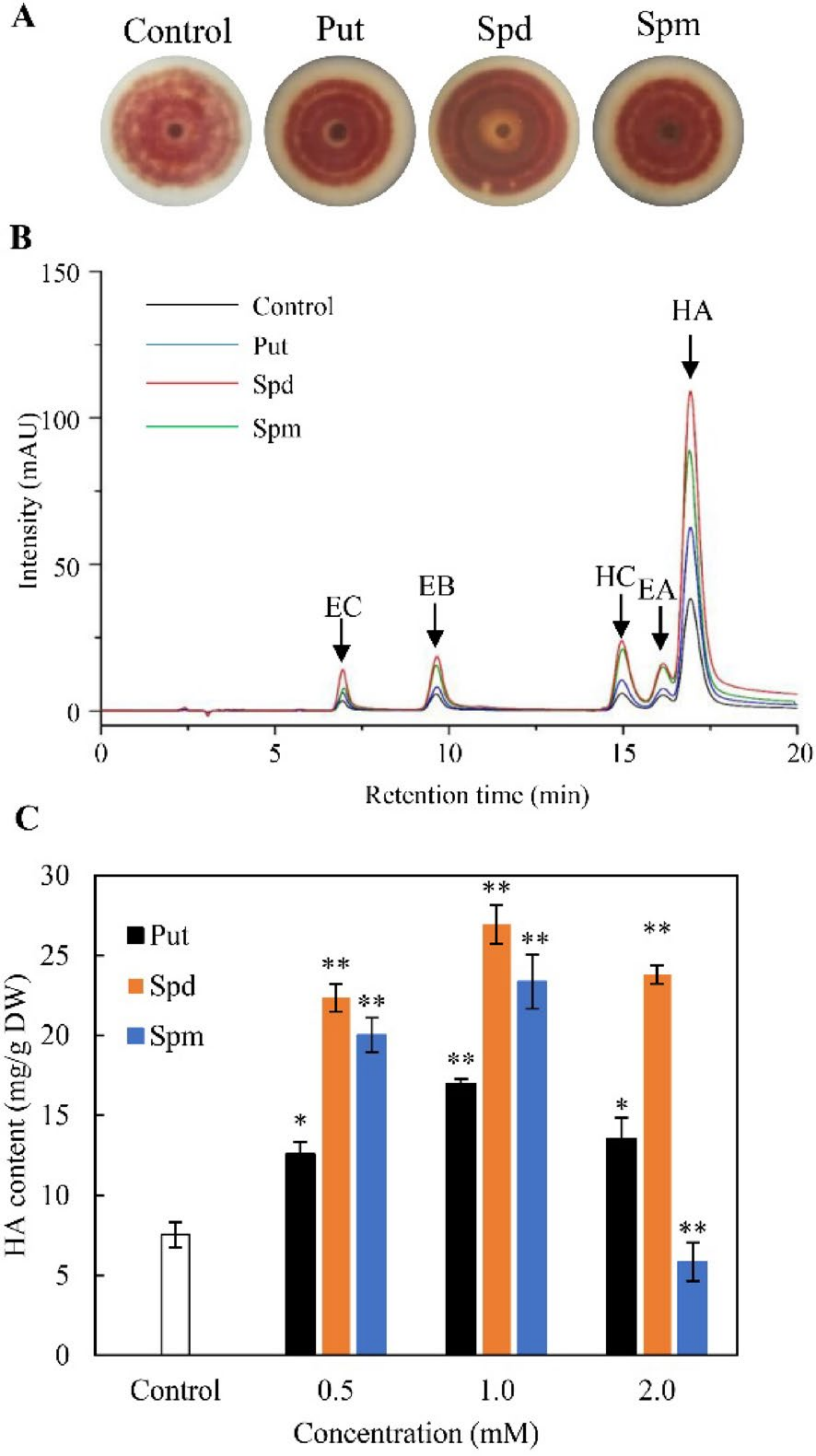
Effects of exogenous polyamines on perylenequinone biosynthesis in *Shiraia* sp. S9. **A** Influence of 1 mM putrescine (Put), spermidine (Spd), or spermine (Spm) on the secretion of red pigments by *Shiraia* sp. S9 cultured on PDA plates. **B** Representative HPLC profiles showing the accumulation of individual perylenequinone compounds. **C** Impact of polyamine supplementation on hypocrellin A (HA) production in submerged mycelial cultures of *Shiraia* sp. S9. Polyamines were introduced on day 4 of cultivation, and untreated cultures served as controls. Fermentations were carried out at 28 °C with shaking at 150 rpm for a total of 8 days. Data are presented as mean ± SD of three independent experiments. Statistical significance was defined as *p* < 0.05 (^*^) and *p* < 0.01 (^**^) compared with the control

To investigate the effects of exogenous polyamines on *Shiraia* sp. S9, PDA medium was supplemented with 1.0 mM of Put, Spd or Spm. All three polyamines significantly enhanced the accumulation of red pigments in solid cultures (Fig. 1A). Specifically, Put, Spd, and Spm markedly increased the production of individual perylenequinones, including HA, HC, EA, EB and EC, with the most pronounced effect observed for HA (Fig. 1B, Table S2). In liquid mycelium cultures supplemented with the polyamine (0.5–2.0 mM) on day 4, maximum HA stimulation occurred at 1.0 mM of Spd while HA reached 26.9 mg/g dry weight (DW), about 3.3-fold over the control (Fig. 1C).

**Fig. 2:**
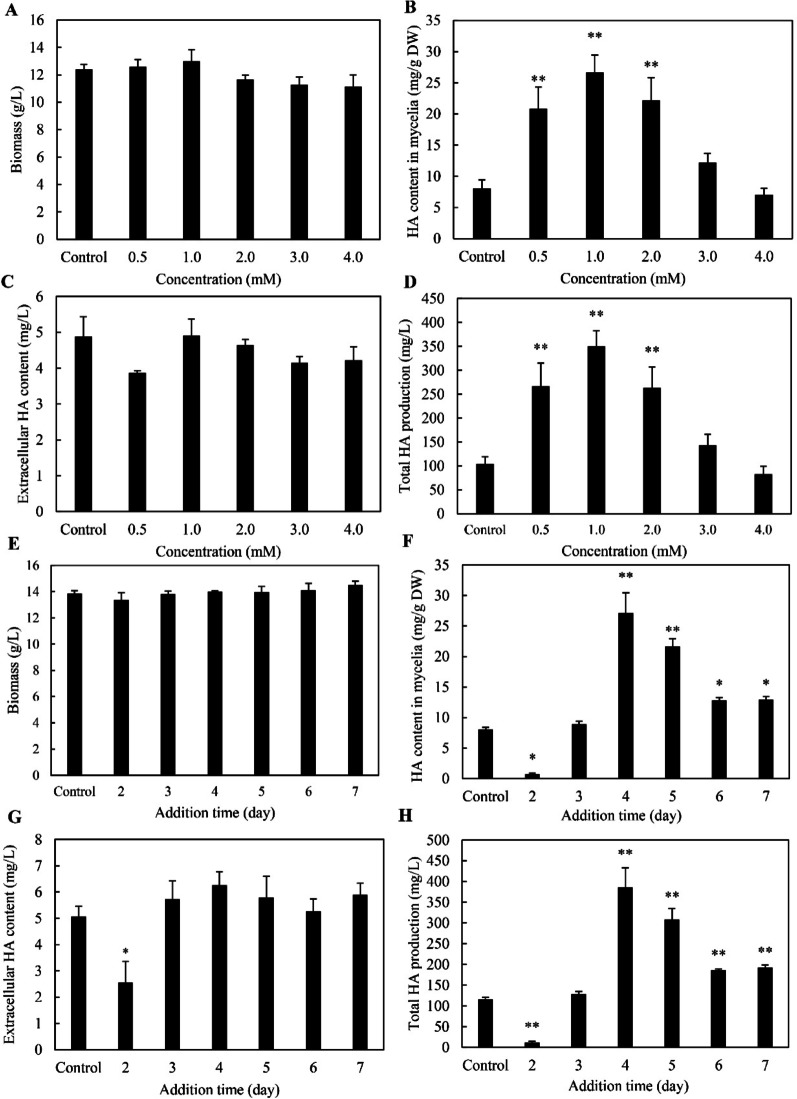
Influence of spermidine (Spd) concentration and application timing on fungal growth and hypocrellin A (HA) biosynthesis in *Shiraia* sp. S9. Effects of varying Spd concentrations on mycelial biomass (**A**), intracellular HA accumulation (**B**), extracellular HA levels in the culture broth (**C**), and total HA yield (**D**). In these experiments, Spd was supplemented on day 4 of cultivation. Effects of different Spd addition time points (1.0 mM) on biomass formation (**E**), intracellular HA content (**F**), HA secretion into the broth (**G**), and overall HA production (**H**). Cultures were incubated at 28 °C with shaking at 150 rpm for 8 days. Untreated cultures served as controls. Data are expressed as mean ± SD from three independent experiments. Statistical significance was defined as *p* < 0.05 (^*^) and *p* < 0.01 (^**^) compared with the control group

To further investigate the impact of Spd on HA production in *Shiraia* sp. S9 mycelium cultures, we supplemented 4-day-old cultures with Spd (0.5–4.0 mM). While Spd did not significantly alter fungal biomass (Fig. 2A), it elicited intracellular HA accumulation at lower concentrations (0.5–1.0 mM), peaking at 1.0 mM (Fig. 2B). Extracellular HA remained unaffected across all concentrations (Fig. 2C), yielding maximal total HA production at 1.0 mM Spd (Fig. 2D). When Spd at 1.0 mM was supplemented on days 2–7 respectively, fungal biomass remained unaffected at all timepoints (Fig. 2E). However, day 2 addition severely inhibited intracellular and extracellular HA production (Fig. 2F, G). Peak stimulation on total HA production (400 mg/L) was reached under day 4 addition, about 3.4-fold over the control (Fig. 2H). These results demonstrated that exogenous Spd differentially regulated intracellular HA pools without altering growth kinetics, establishing its role as a potent elicitor of perylenequinone biosynthesis in *Shiraia* sp. S9. Subsequent investigations therefore focus on Spd’s physiological mechanisms.

### Effects of Spd and its related metabolites on NO production

**Fig. 3:**
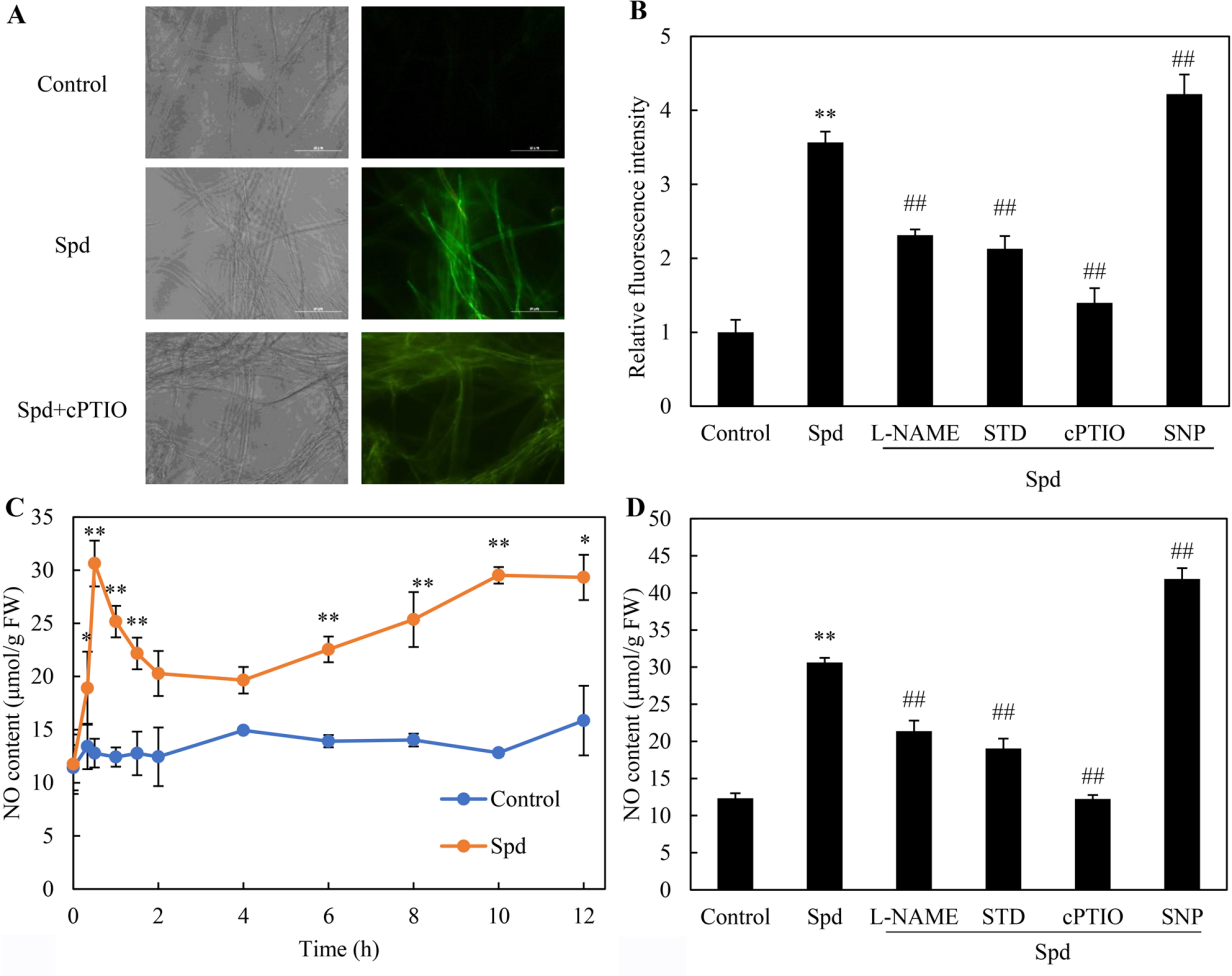
Effects of spermidine (Spd) on nitric oxide (NO) production and the activities of nitric oxide synthase (NOS) and nitrate reductase (NR) in *Shiraia* sp. S9. **A** Visualization of intracellular NO in S9 mycelia using DAF-2 DA staining. Representative bright-field images (left) and corresponding fluorescence micrographs (right) are shown (400 × magnification). **B** Quantification of NO accumulation based on relative fluorescence intensity. **C** Temporal profile of NO production in mycelia following Spd exposure. **D** Intracellular NO levels under different pharmacological treatments. Spd (1.0 mM) was administered on day 4 of cultivation. The NO donor sodium nitroprusside (SNP, 5 μM) and the inhibitors L-NAME (100 μM), sodium tungstate dihydrate (STD, 100 μM), and cPTIO (100 μM) were applied 30 min prior to Spd addition. Microscopic observations were performed 30 min after Spd treatment. Cultures without Spd supplementation served as controls. Data are presented as mean ± SD from three independent experiments. Statistical differences were considered significant at *p* < 0.05 (^*^) and *p* < 0.01 (^**^) compared with the control, and at *p* < 0.01 (^##^) compared with the Spd-treated group

Spd treatment (1.0 mM) induced significant NO production in hyphae, visualized as intensified green fluorescence using the NO-specific probe DAF-2 DA (Fig. 3A). This signal was attenuated by pretreatment with the NOS inhibitor L-NAME (100 μM), the NR inhibitor STD (100 μM), or the NO scavenger cPTIO (100 μM), while the NO donor SNP (5 μM) amplified the response (Fig. 3B). NO levels peaked 30 min after Spd treatment, reaching a 2.4-fold increase relative to controls (Fig. 3C). The pretreatment with L-NAME, STD, and cPTIO reduced Spd-induced NO generation by 30.2%, 37.8%, and 60.0%, respectively, whereas SNP enhanced NO content by 30.7% compared to Spd treatment alone (Spd + SNP vs. Spd in Fig. 3D).

**Fig. 4:**
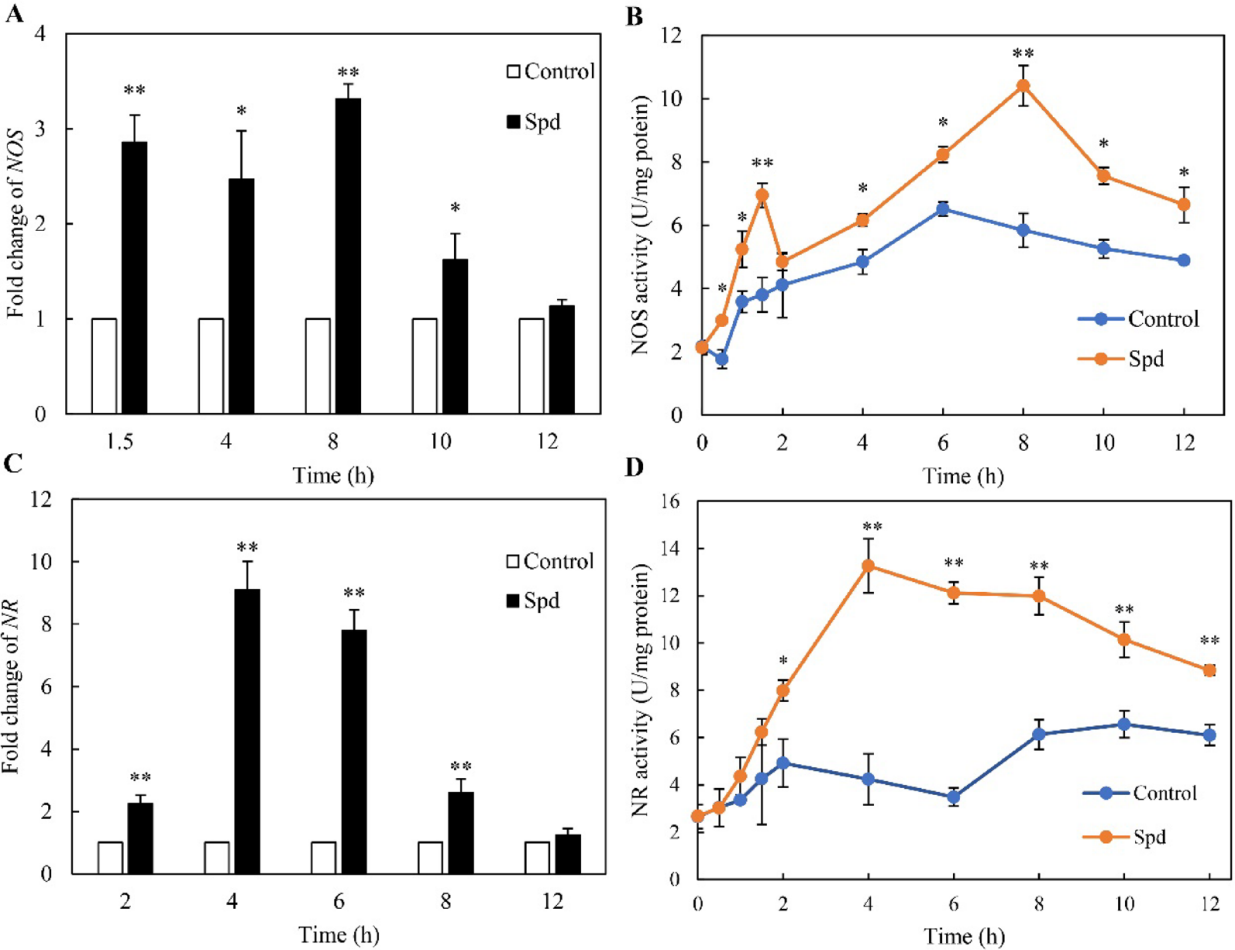
Effects of spermidine (Spd) on gene expression (**A**) and activities of nitric oxide (NOS) (**B**), and the expression of nitrate reductase (NR) gene (**C**) and its activities (**D**) of *Shiraia* sp. S9. Spd (1 mM) was added on day 4 of the mycelium culture. Values are mean ± SD from three independent experiments (^*^p < 0.05 and ^**^p < 0.01 versus control)

**Fig. 5:**
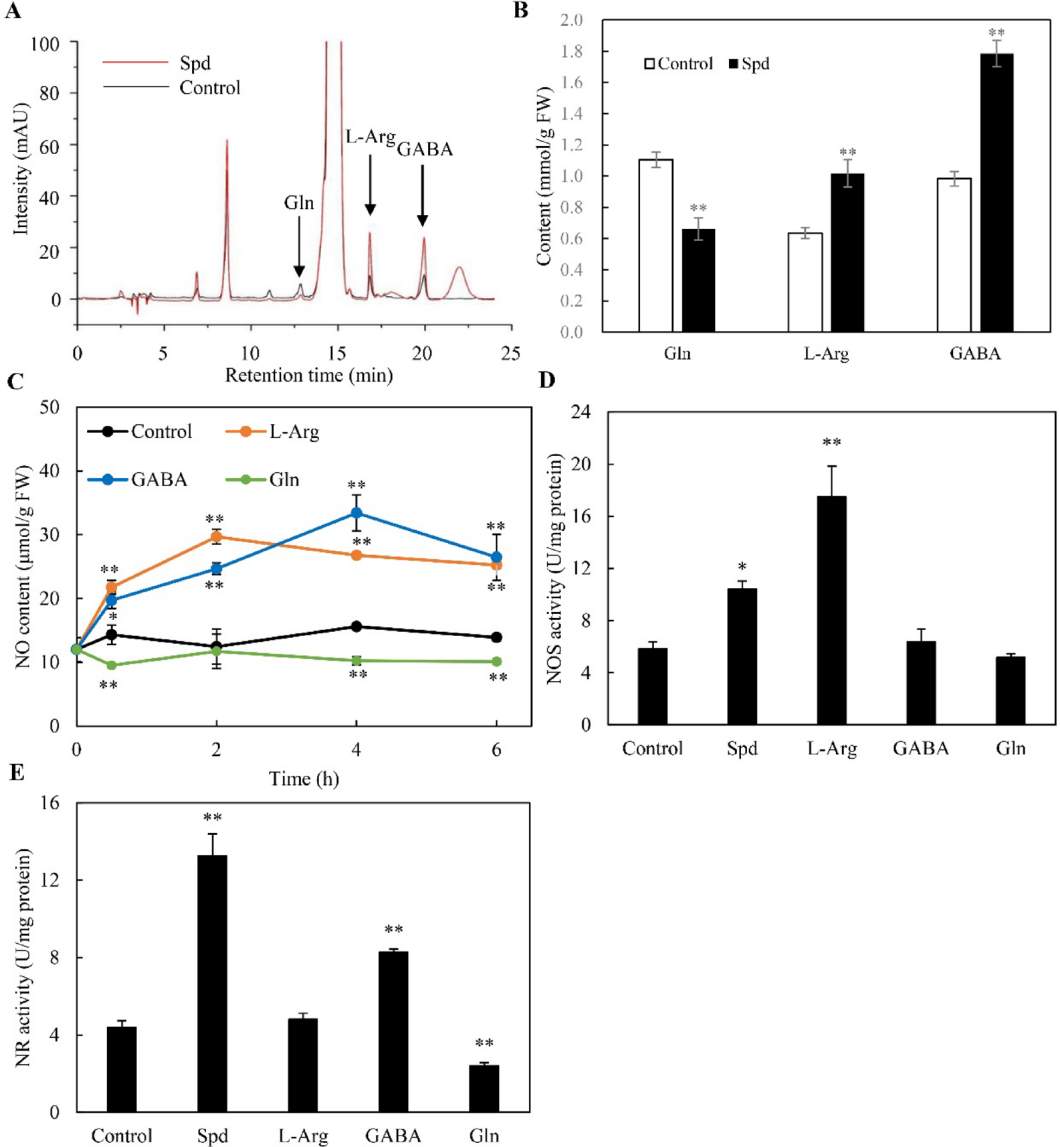
Effects of spermidine (Spd) on endogenous glutamine (Gln), L-arginine (L-Arg) and γ-aminobutyric acid (GABA) of *Shiraia* sp. S9. **A** Chromatogram of Gln, L-Arg and GABA in mycelia after Spd treatment. **B** The content of Gln, L-Arg and GABA in mycelia after Spd treatment. Effects of exogenous Gln, L-Arg and GABA on NO generation **C**, nitric oxide synthase (NOS) activity (**D**) and nitrate reductase (NR) activity (**E**). Spd, Gln, L-Arg or GABA was added at 1.0 mM receptively on day 4 of the culture. The culture was maintained at 150 rpm and 28℃ for 8 days. Values are mean ± SD from three independent experiments (^*^p < 0.05 and ^**^p < 0.01 versus control)

Further analysis showed that Spd upregulated the gene expression and enzymatic activities of both NOS (Fig. 4A, B) and NR (Fig. 4C, D). These coordinated transcriptional and enzymatic responses demonstrated that Spd activated both NOS- and NR-mediated pathways for NO biosynthesis in *Shiraia* sp. S9. Spd can be metabolized into L-Arg, GABA, and Gln through enzymatic reactions to regulate NO production in plants (Recalde et al. 2021). Herein, we measured endogenous contents of these metabolites after Spd treatment (1.0 mM, day 4). Spd treatment significantly elevated endogenous L-Arg and GABA concentrations in *Shiraia* hyphae after 4 days of culture, while reducing Gln levels (Fig. 5A, B). Exogenous L-Arg and GABA supplementation elevated NO production throughout 0–6 h, whereas Gln consistently suppressed NO generation (Fig. 5C). After 8 h of 1 mM L-Arg treatment, NOS activity was enhanced to 17.4 U/mg protein, 3.0-fold higher than controls, while GABA and Gln showed no significant effects (Fig. 5D). Conversely, GABA increased NR activity to 8.3 U/mg protein, 1.9-fold higher than controls at 4 h, and Gln reduced NR activity, with L-Arg exerting no significant effect on NR (Fig. 5E). These findings demonstrated distinct regulatory mechanisms of Spd metabolites: L-Arg primarily modulated NO production through NOS activation, while GABA and Gln influenced NO synthesis via NR-dependent pathways.

### Effects of NO and sGC-cGMP pathway on HA production

**Fig. 6:**
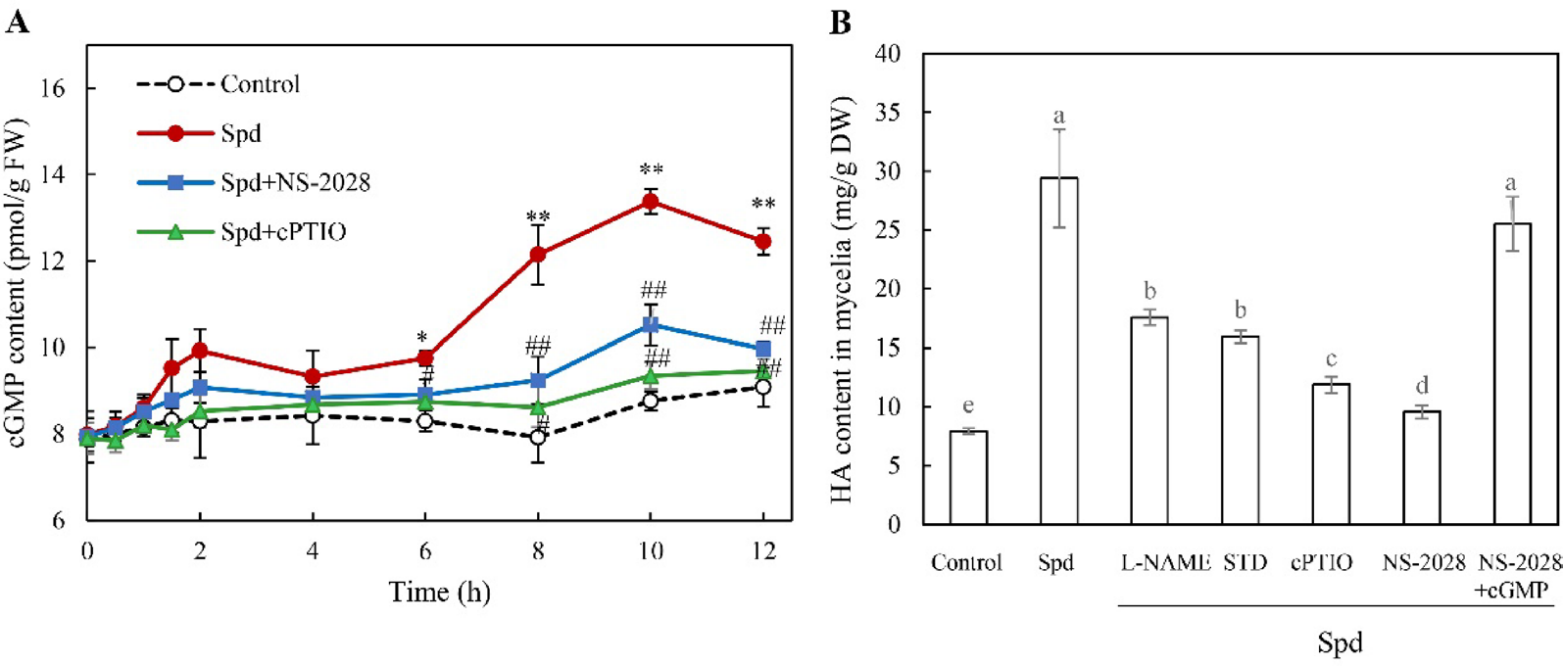
Effects of spermidine (Spd) treatment on cGMP content and HA production in *Shiraia* sp. S9. **A** Time course of cGMP content after Spd treatment. **B** Effects of Spd and the induced NO generation on HA production. Spd was added at 1.0 mM on day 4 of the culture and the culture was maintained at 150 rpm and 28℃ for 8 days. L-NAME (100 μM), STD (100 μM), cPTIO (100 μM), NS-2028 (10 μM) and cGMP (5 mM) were added to the culture 30 min prior to Spd treatment. The culture without Spd treatment was used as the control. Values are mean ± SD from three independent experiments (^*^p < 0.05 and ^**^p < 0.01 versus control, ^#^*p* < 0.05 and ^##^*p* < 0.01 versus Spd group)

sGC-generated cGMP serves as a critical downstream signaling molecule in NO cascades (Zhao et al. 2020). To investigate Spd’s impact on the NO-sGC-cGMP signaling pathway in *Shiraia* sp. S9, we quantified cGMP in hyphae following Spd treatment (1.0 mM, day 4) with or without pretreatment using the sGC inhibitor NS-2028 (Fig. 6). Spd treatment significantly elevated intracellular cGMP accumulation after 4 h. Pretreatment with the sGC inhibitor NS-2028 reduced cGMP content by 27.1% compared to Spd treatment alone. The increased cGMP by Spd treatment was mitigated further in the presence of the NO scavenger cPTIO (Fig. 6A). Furthermore, the pretreatment with the NOS inhibitor L-NAME (100 μM), NR inhibitor STD (100 μM), NO scavenger cPTIO (100 μM), or sGC inhibitor NS-2028 (10 μM) inhibited HA yields by 40.3%, 45.8%, 59.6%, and 67.5%, respectively, relative to Spd treatment alone (Fig. 6B). However, exogenous cGMP (5 mM) rescued HA biosynthesis under the condition where sGC was inhibited by NS-2028. These results indicated that the Spd-elicited enhancement of HA biosynthesis is mediated by the NO–sGC–cGMP signaling cascade.

### Transcriptomic profiling and metabolic regulation

**Fig. 7:**
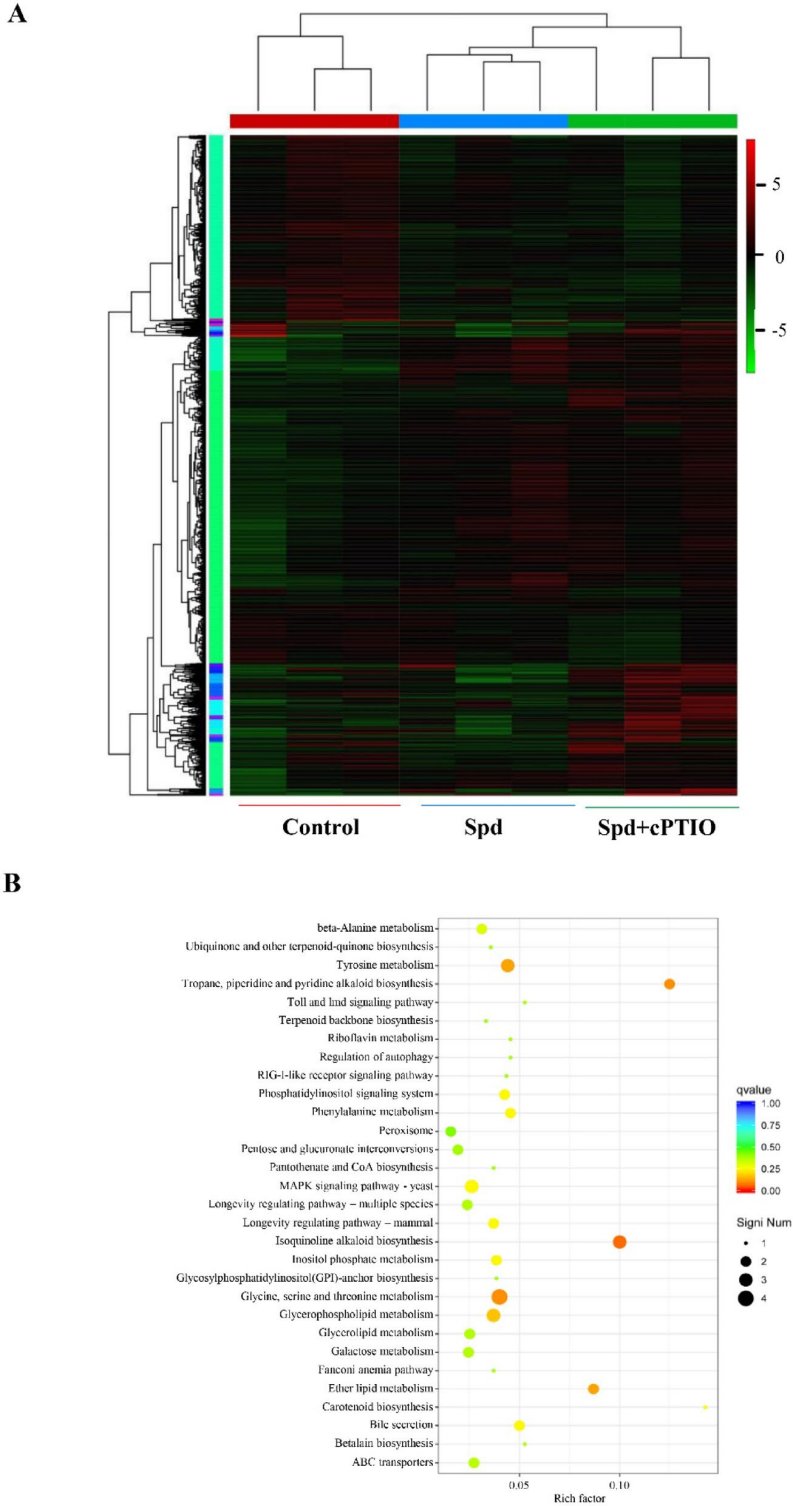
Heatmap (**A**) and KEGG pathways (**B**) of differentially expressed genes (DEGs) of *Shiraia* sp. S9 after spermidine (Spd) treatment. Spd was added at 1 mM on day 4 of the culture and the culture was maintained at 150 rpm and 28℃ for 7 days. cPTIO (100 μM) was added to the culture 30 min prior to Spd treatment. Cultures without Spd supplementation served as controls

**Fig. 8:**
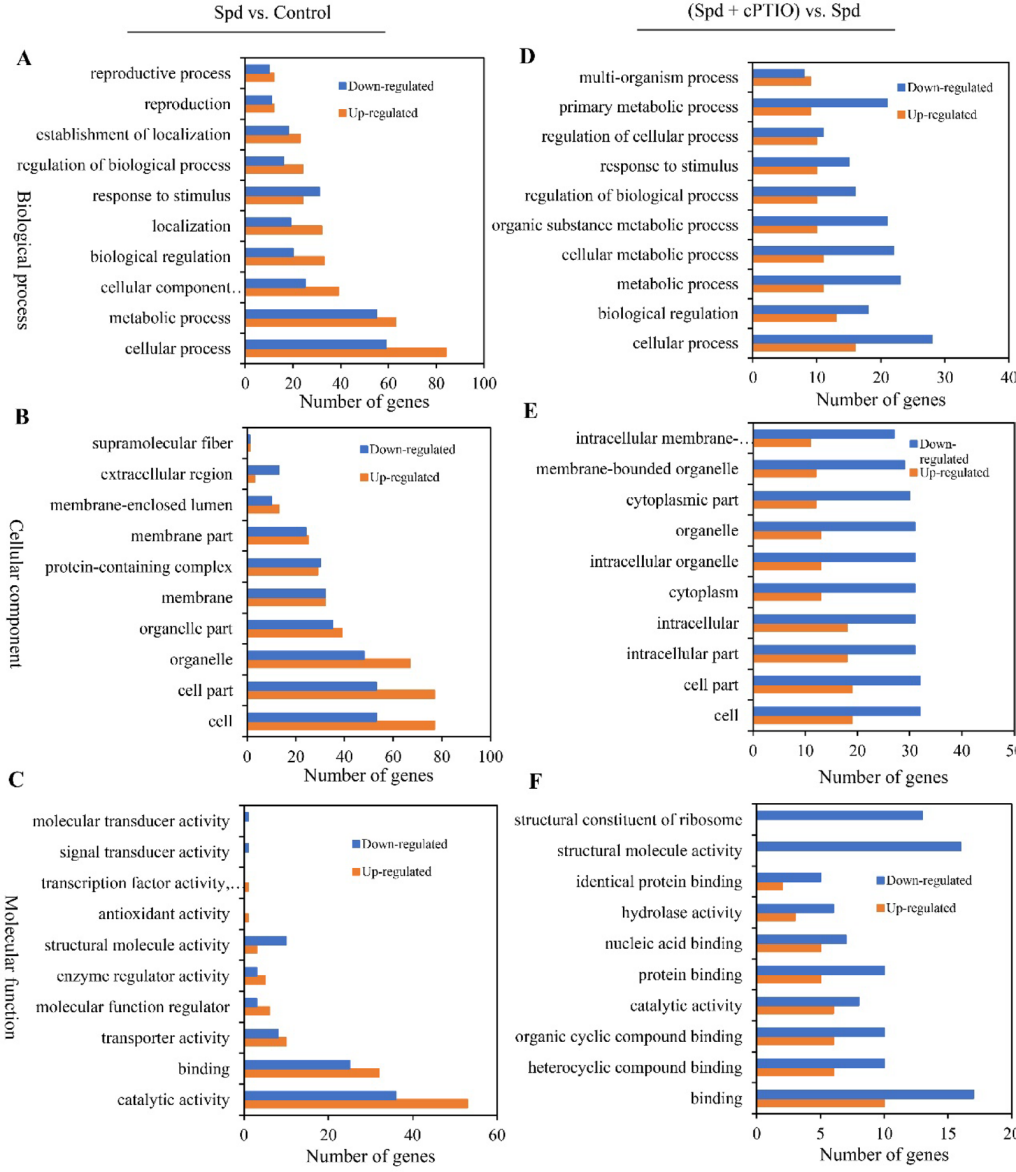
Gene ontology (GO) classification of differentially expressed genes (DEGs) under spermidine (Spd) vs. control (left) and Spd treatment + cPTIO vs. control (right). The results are classified in three main categories: biological process (**A**, **D**), cellular component (**B**, **E**) and molecular function (**C**, **F**). Spd (1.0 mM) was added on day 4 and cPTIO (100 μM) was added 30 min prior to Spd treatment. The culture was maintained at 28 °C for 7 days

To elucidate NO-dependent mechanisms underlying Spd-induced HA biosynthesis, RNA sequencing was performed on *Shiraia* sp. S9 hyphae treated with 1.0 mM Spd and the combination of 1.0 mM Spd with 100 μM NO scavenger cPTIO (Spd + cPTIO). Following RNA extraction and cDNA library construction, high-throughput sequencing generated 53.5–70.1 million total clean reads per sample after quality control. The mass values Q20 and Q30 were all above 95% (Table S3). De novo assembly yielded 57,069 transcripts and 29,858 unigenes with N50 values of 6,913 bp and 3,498 bp, respectively (Fig. S1). To further investigate on the differentially expressed genes (DEGs), the transcripts with log_2_|fold change||≥ 1 and a *P* value ≤ 0.05 in each biological replicate were identified. There were 587 differentially expressed genes (DEGs) in Spd-treated groups versus controls (310 upregulated, 277 downregulated), while Spd + cPTIO treatment produced 266 DEGs relative to Spd treatment alone (55 upregulated, 211 downregulated) (Figs. 7A, S2). KEGG pathway enrichment revealed that Spd significantly upregulated DEGs associated with carbohydrate metabolism including glycolysis, the tricarboxylic acid (TCA) cycle, pentose/glucuronate interconversions, and amino acid metabolism (glycine/serine/threonine, phenylalanine/tyrosine pathways), which are involved in perylenequinone biosynthesis (Fig. 7B). Some DEGs were annotated into signal transduction pathways (MAPK and phosphatidylinositol signaling), indicating the possible signaling roles of NO induced by Spd. Furthermore, GO enrichment analysis showed Spd-induced DEGs were predominantly enriched in biological processes (BP) including cellular organization (GO:0016043) and metabolic regulation (GO:0008152), cellular components (CC) such as organelles (GO:0043226) and membranes (GO:0016020), and molecular functions (MF) including catalytic activity (GO:0003824) and transporter regulation (GO:0030234) (Fig. 8A, B, C). cPTIO co-treatment reversed these enrichments, downregulating genes involved in intracellular organization (GO:0044424), heterocyclic compound binding (GO:0001803), and metabolic regulation processes relative to Spd treatment (Fig. 8D, E, F), confirming NO as a regulator of these metabolic pathways.

### The mediation of NO in Spd-induced HA biosynthesis

**Fig. 9:**
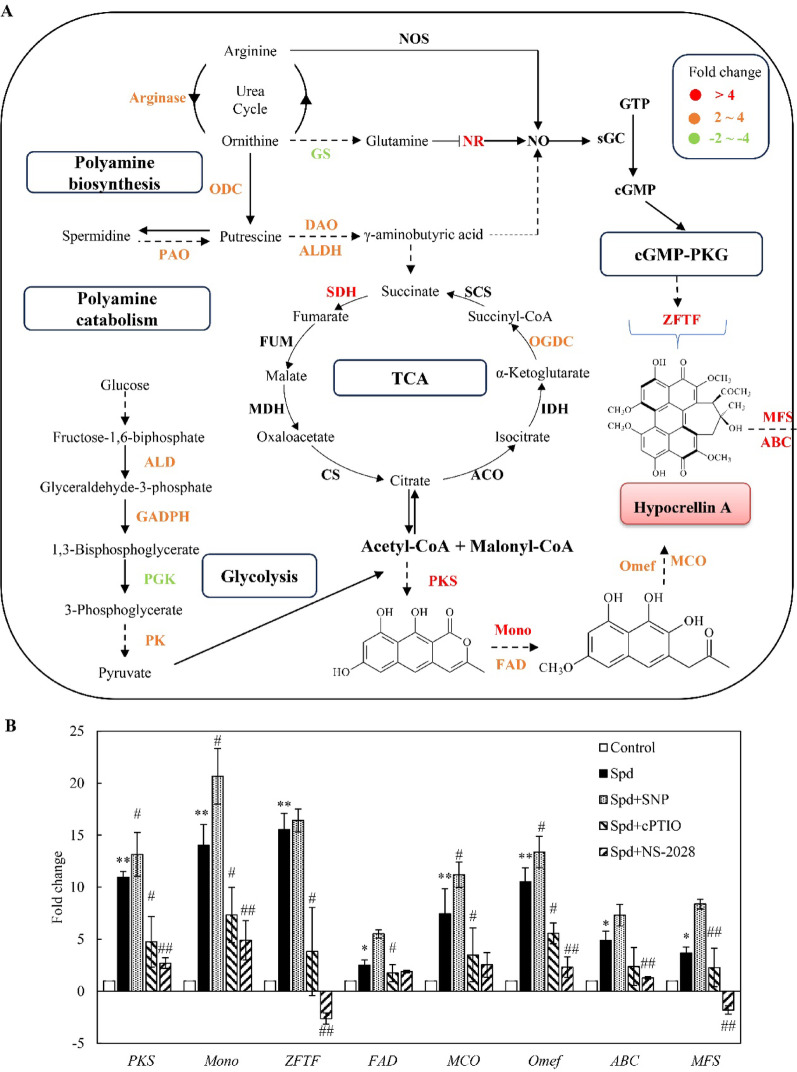
Effects of spermidine (Spd) on gene expressions for HA biosynthesis in *Shiraia* sp. S9. **A** A schematic diagram of Spd-induced HA biosynthesis of *Shiraia* sp. S9. Genes shown in red represent the up-regulated genes, and green represents the down-regulated genes. Some of steps and compounds are omitted for simplification. The dotted arrows indicate more uncertain steps. TCA tricarboxylic acid cycle, PKS polyketide synthase, Mono monooxygenase, ZFTF zinc finger transcription factor, FAD FAD/FMN-dependent oxidoreductase, Omef *O*-methyltransferase, MCO multicopper oxidase, MFS major facilitator superfamily, ABC ATP-binding cassette transporter, NO nitric oxide, NOS nitric oxide synthase, sGC soluble guanylate cyclase. GTP guanosine triphosphate, PKG protein kinase G. More information about enzyme and annotations are given in Table S4. **B** The Spd-induced expressions of key genes for HA biosynthesis. Spd was added at 1 mM on day 4. SNP (5 μM), L-NAME (100 μM), STD (100 μM), cPTIO (100 μM) or NS-2028 (10 μM) was added respectively to the culture 30 min prior to Spd treatment. The culture without Spd treatment was used as control. The culture was incubated at 150 rpm and 28℃. Values are mean ± SD from three independent experiments (^*^p < 0.05 and ^**^p < 0.01 versus control, ^#^*p* < 0.05 and ^##^*p* < 0.01 versus Spd group)

Glycolysis and TCA cycle provide essential carbon precursors for perylenequinone biosynthesis. Following Spd treatment, gene expressions in the glycolytic pathway was significantly upregulated (2.1- to 3.4-fold), including fructose-1,6-bisphosphate aldolase (ALD, TRINITY_DN14363_c0_g1), glyceraldehyde-3-phosphate dehydrogenase (GAPDH, TRINITY_DN2339_c0_g1), pyruvate kinase (PK, TRINITY_DN18781_c0_g4), phosphoglycerate mutase (PGAM, TRINITY_DN29954_c0_g1), alcohol dehydrogenase (ADH, TRINITY_DN19181_c0_g3), and lactate dehydrogenase (LDH, TRINITY_DN2275_c0_g1) genes, while downregulating phosphoglycerate kinase (PGK, TRINITY_DN3463_c0_g1) and enolase (ENO, TRINITY_DN16773_c0_g2) genes (Table S4, Fig. 9A). Furthermore, Spd induced significant upregulation of TCA cycle genes encoding succinate dehydrogenase (SDH, TRINITY_DN19976_c0_g1) and the α-ketoglutarate dehydrogenase complex (OGDC, TRINITY_DN24250_c0_g1). Notably, pretreatment with NO scavenger cPTIO substantially shifted these expression patterns, reducing transcript levels of all aforementioned genes relative to Spd treatment alone. These findings collectively indicate enhanced central carbon metabolic flux during Spd-induced HA biosynthesis and establish NO as a critical regulator of glycolytic and TCA pathway activation.

Based on the genome sequencing of *Shiraia* strains (Yang et al. 2014; Ren et al. 2020), we identified 26 genes significantly annotated to the hypocrellin biosynthetic gene cluster. Transcriptomic analysis confirmed coordinated induction of putative polyketide synthase (PKS, TRINITY_DN3431_c0_g1), conidial yellow pigment biosynthesis PKS (TRINITY_DN16335_c0_g1), *O*-methyltransferase (Omef, TRINITY_DN13569_c0_g1), FAD-binding monooxygenase (Mono, TRINITY_DN15905_c0_g1), zinc finger transcription factor (ZFTF, TRINITY_DN18687_c0_g4), multicopper oxidase (MCO, TRINITY_DN18528_c0_g1), FAD-dependent oxidoreductase (FAD, TRINITY_DN3307_c0_g1), isotrichodermin C-15 hydroxylase (TRINITY_DN30003_c0_g1), major facilitator superfamily (MFS) transporter (TRINITY_DN20192_c0_g1) and ATP-binding cassette transporter (ABC) transporter (TRINITY_DN16243_c0_g2) genes (Table S4, Fig. 9A). Scavenging Spd-induced NO with cPTIO attenuated these responses. Expression of *PKS* (TRINITY_DN28558_c0_g1), flavin-binding monooxygenase (TRINITY_DN23121_c0_g1), *ZFTF* (TRINITY_DN18687_c0_g4) and *MFS* (TRINITY_DN20192_c0_g1) reverted to control levels, while *Mono* (TRINITY_DN4415_c0_g1) and *PKS* (TRINITY_DN13421_c0_g1) became significantly downregulated. Quantitative RT-PCR confirmed Spd-mediated upregulation of HA biosynthetic genes from 2.5- to 15.5-fold increases versus controls (Fig. 9B). To elucidate role of NO in Spd*-*induced HA biosynthesis in *Shiraia* sp. S9, mycelium cultures were pre-incubated with NOS inhibitor L-NAME, NR inhibitor STD, NO scavenger cPTIO, or sGC inhibitor NS-2028, respectively. cPTIO pretreatment reversed these inductions, reducing *PKS*, *Mono*, *ZFTF*, *FAD*, *MCO*, *Omef* and *MFS* expression to 4.8-, 7.3-, 3.8-, 1.8-, 2.6-, 5.6-, and 2.3-fold of Spd treatment alone. Conversely, the NO donor SNP further enhanced *PKS*, *Mono*, *MCO*, and *Omef* expression to 13.2-, 20.7-, 11.2-, and 13.4-fold above controls. sGC inhibitor NS-2028 further suppressed Spd-induced expression of most HA biosynthetic genes, with *ZFTF* and *MFS* exhibiting significant downregulation, though *FAD* and *MCO* remained unaffected (Fig. 9B). These results provide additional evidence that Spd regulated HA biosynthesis through NO-dependent sGC-cGMP signaling.

### Combined elicitation of Spd and SNP on *Shiraia* HA production

**Fig. 10:**
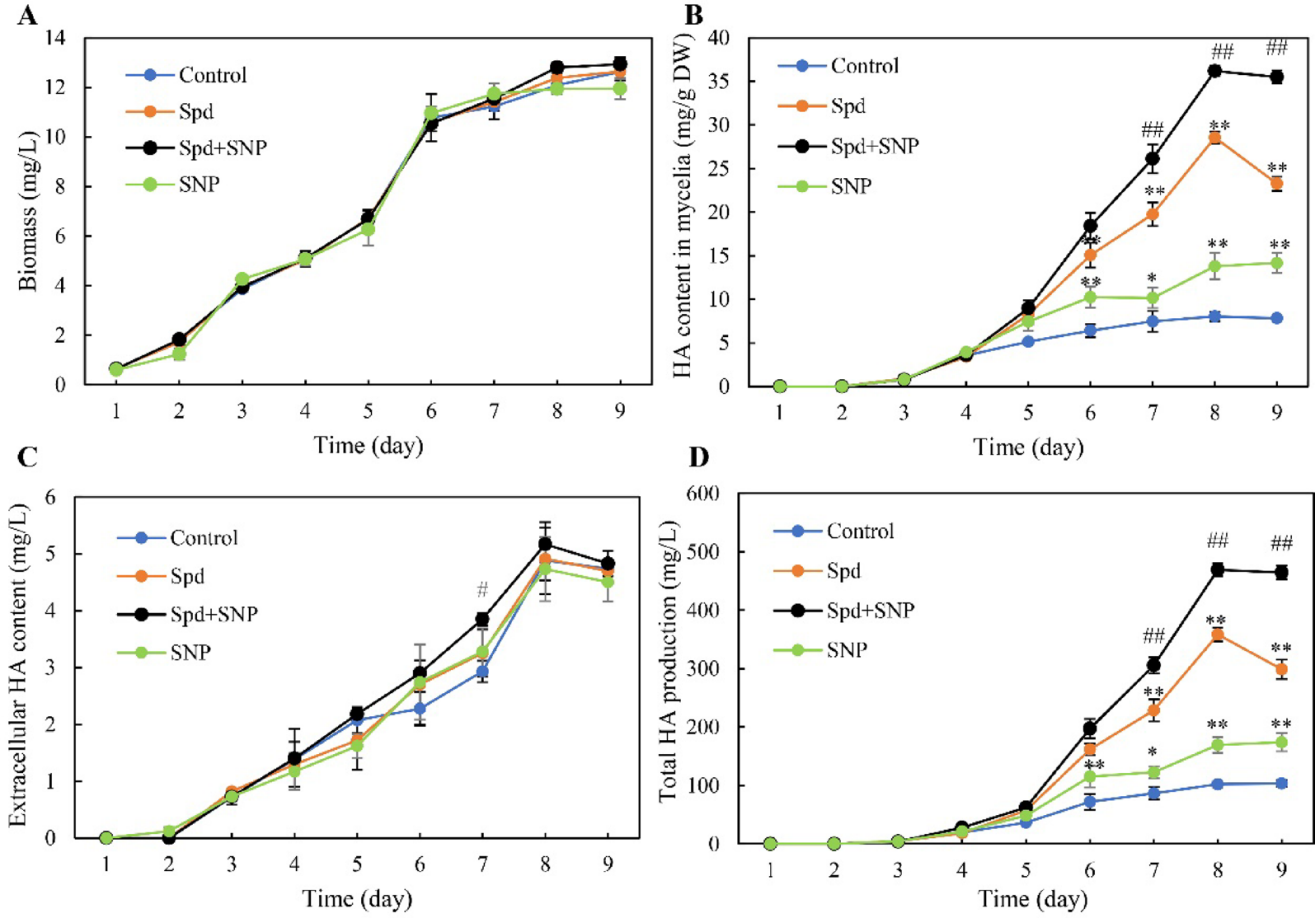
Time-course analysis of spermidine (Spd) effects on growth and hypocrellin A (HA) production in *Shiraia* sp. S9. **A** Mycelial biomass; **B** intracellular HA concentration; **C** extracellular HA accumulated in the culture broth; and **D** total HA yield over the cultivation period. Spd (1.0 mM) was supplemented on day 4 of fermentation. The nitric oxide donor sodium nitroprusside (SNP, 5.0 μM) was administered 30 min prior to Spd treatment where indicated. Cultures were incubated at 28 °C with shaking at 150 rpm. Untreated cultures served as controls. Data are expressed as mean ± SD from three independent biological replicates. Statistical significance was defined as ^**^p < 0.01 compared with the control, and ^#^*p* < 0.05 or ^##^*p* < 0.01 compared with the Spd-treated group

Based the expression analysis of NO effects on HA biosynthesis, we want to further enhance the HA production by using NO donor SNP. Although treatment with SNP alone didn’t suppressed the growth of *Shiraia* sp. S9 (Fig. 10A), it significantly stimulated mycelial HA biosynthesis (Fig. 10B). However, extracellular HA accumulation was not markedly enhanced by SNP under the same conditions (Fig. 10C). Following 4 days of exposure to 5.0 μM SNP, HA production reached 169.5 mg/L, approximately 1.7-fold higher than that of the control (Fig. 10D). Then, we introduced SNP (1.0–20.0 μM) in the culture 30 min prior to 1 mM Spd treatment. Although SNP at 1.0–20.0 μM revealed no significant differences in *Shiraia* biomass (Fig. S3A), co-application of Spd at 1.0 mM with 5.0 or 10.0 μM SNP significantly enhanced intracellular HA production (Fig. S3B). Extracellular HA remained unaffected across all concentrations of the applied SNP (Fig. S3C). The highest HA production was achieved under 5.0 μM SNP and 1.0 mM Spd treatment (Fig. S3D). Temporal optimization demonstrated that SNP supplementation timing critically modulated efficacy (Fig. S4): addition of 5.0 μM SNP on day 4 followed by 1.0 mM Spd (30 min later) yielded maximal intracellular HA enhancement, while later applications progressively diminished this effect. Under this optimized condition, fungal biomass increased rapidly during days 2–6 followed by gradual rise after day 7, with no intergroup differences between the treatment of Spd or Spd + SNP (Fig. 10A). The intracellular HA accumulation increased significantly from day 5, peaking at 36.2 mg/g DW on day 8 (Fig. 10B). Extracellular HA patterns mirrored control and Spd-only groups without significant alterations (Fig. 10C). Total HA production reached 469.2 mg/L by day 8, corresponding to 4.6-fold and 1.3-fold enhancements over control and Spd-only treatments (Fig. 10D).

## Discussion

Fungal perylenequinones and their derivatives play crucial ecological roles as virulence factors and defense metabolites during host–pathogen interactions. In phytopathogenic fungi such as *S. bambusicola, Cercospora beticola*, and *Elsinoë fawcettii*, the photoactivated perylenequinones act as secondary metabolites mediating light-dependent ROS generation, which promote infection, necrosis, and colonization in plant tissues (Daub et al. 2013). In *Shiraia* species, hypocrellins represent the major perylenequinones with strong photodynamic activity, whose biosynthesis is highly sensitive to environmental factors such as light–dark cycles, pH, carbon/nitrogen sources, and various abiotic or biotic elicitors (Li et al. 2025). Recent studies have revealed that several signaling molecules-including ROS, calcium ions (Ca^2^⁺), NO and mitogen-activated protein kinase (MAPK) pathways mediated the elicitation of hypocrellin biosynthesis in *Shiraia* mycelium cultures (Lu et al. 2019; Chen et al. 2022; Li et al. 2024). In the present study, we identified polyamines, particularly Spd, as a novel category of endogenous signaling regulators that markedly stimulate HA biosynthesis via NO signaling. In plants, polyamines such as Put, Spd, and Spm are well-recognized signaling molecules that regulate growth, stress tolerance, and secondary metabolism through NO-dependent cascades (Recalde et al. 2021). However, evidence for NO involvement in polyamine-mediated regulation of fungal metabolism has been scarce. Although NO has been implicated as a developmental and metabolic signal in filamentous fungi (Zhao et al. 2020), the current work demonstrates for the first time that polyamines can trigger NO generation and activate the downstream sGC–cGMP signaling pathway to induce secondary metabolite biosynthesis in fungi. This finding highlights a previously unrecognized signaling mechanism for perylenequinone regulation and broadens our understanding of NO-mediated polyamine signaling beyond the plant kingdom.

NO is a small, diffusible gaseous signaling molecule with multifaceted functions in fungi, including morphogenesis, sporulation, stress responses, and secondary metabolite regulation (Zhao et al. 2020). In filamentous fungi, NO can be synthesized through two major enzymatic routes: NOS-dependent oxidation of L-arginine and the NR-mediated reduction of nitrite (Turrion-Gomez and Benito 2011). The relative contributions of these pathways vary among species and physiological conditions. For example, the NR-mediated route predominates in *Neurospora crassa* and *Aspergillus nidulans*, while both NOS-like and NR activities contribute to NO formation in *Botrytis cinerea* and *Fusarium graminearum* (Baidya et al. 2011; Golderer et al. 2001). In *Shiraia sp.* S9, our results demonstrated that exogenous Spd triggered rapid and sustained NO accumulation mediated by both NOS- and NR-dependent pathways (Fig. 3). The enhanced gene expression and enzymatic activities of NOS and NR after Spd treatment, and their significant suppression by inhibitors (L-NAME and STD), indicated dual enzymatic contributions to Spd-induced NO production. In plants, polyamines are closely linked with NO biosynthesis and its signaling. L-Arginine, a central intermediate of polyamine metabolism, serves as a precursor for NOS-dependent NO formation, while catabolic products such as GABA and hydrogen peroxide may also modulate NR-dependent NO generation (Tun et al. 2006; Recalde et al. 2021). Spd-induced NO production in *Arabidopsis thaliana* and tobacco cells is known to mediate stomatal closure, oxidative stress tolerance, and the activation of defense genes (Recalde et al. 2021). Our study reveals a similar mechanistic link in *Shiraia*, where Spd metabolism elevated endogenous L-Arg and GABA levels, each associated with increased NOS and NR activities, respectively. This finding suggests that fungal polyamine catabolism follows analogous biochemical logic to plants, integrating L-Arg- and GABA-dependent pathways for NO generation. Although previous studies in fungi have described NO as a developmental or stress-related signal (Baidya et al. 2011; Zhao et al. 2020), direct evidence linking polyamines to NO generation and secondary metabolism was lacking. The current work provides the first demonstration that polyamines can serve as upstream inducers of NO biosynthesis in fungi through dual enzymatic systems. This dual-pathway activation represents a novel regulatory paradigm for fungal secondary metabolism and highlights evolutionary convergence between fungal and plant NO signaling networks under polyamine regulation.

NO often functions as a versatile intracellular messenger, and one of its key transduction routes involves activation of sGC to generate cGMP, a secondary messenger that modulates downstream gene expression and metabolism. In filamentous fungi, the NO-sGC-cGMP signaling cascade has been implicated in regulating developmental differentiation, oxidative stress responses, and secondary metabolite production (Simontacchi et al. 2013; Zhao et al. 2020). In *Shiraia sp.* S9, our study demonstrated that Spd-induced NO production significantly elevated intracellular cGMP levels, which were suppressed by the sGC inhibitor NS-2028 and NO scavenger (cPTIO), confirming the activation of this pathway (Fig. 6). The inhibition of HA accumulation by NOS and NR inhibitors, NO scavenger (cPTIO), and sGC blockade indicated that the Spd-elicited enhancement of HA biosynthesis was mediated primarily through the NO–sGC–cGMP cascade. Transcriptomic data further supported this conclusion: Spd treatment upregulated genes involved in glycolysis and TCA cycle, all providing carbon skeletons and reducing equivalents for perylenequinone biosynthesis, while NO scavenging reversed these transcriptional responses (Fig. 9).

In fungi such as *A. nidulans* and *N. crassa*, cGMP signaling has been shown to activate MAPK and protein kinase G (PKG) pathways that regulate sporulation and secondary metabolism (Simontacchi et al. 2013; Baidya et al. 2011). A similar mechanism may operate in *Shiraia*, where elevated cGMP likely triggers a kinase-mediated transcriptional activation of the hypocrellin biosynthetic gene cluster. Indeed, our RNA-seq results revealed coordinated upregulation of key genes within the hypocrellin biosynthetic cluster, including the core *PKS*, *Omef*, *FAD*, and *MCO*, along with regulatory transcription factors such as *ZFTF* and *MFS* transporter genes. These inductions were abolished by cPTIO and NS-2028, indicating that NO-derived cGMP acts as a master regulator linking primary metabolism to secondary biosynthetic gene expression. This NO-cGMP control of metabolic activation parallels that observed in plants, where NO signaling modulates phenylpropanoid, alkaloid, and flavonoid biosynthesis through cGMP-dependent protein kinases and transcription factors (Simontacchi et al. 2013). Therefore, the present study provides strong evidence that polyamine-triggered NO signaling in *Shiraia* integrates enzymatic NO biosynthesis with cGMP-mediated transcriptional reprogramming to enhance hypocrellin production. This represents, to our knowledge, the first report of a fungal secondary metabolic pathway regulated through a polyamine–NO–cGMP axis, revealing a signaling mechanism that converges with but functionally extends the plant NO regulatory network into fungal systems.

In the present study, co-application of Spd and NO donor SNP significantly enhanced intracellular HA accumulation by 4.6-fold compared with the control, outperforming the effects of Spd or SNP applied individually. This strong enhancing effect suggests that while Spd acts upstream to trigger endogenous NO biosynthesis through both NOS and NR pathways, SNP supplementation ensures sustained NO availability and amplifies downstream sGC–cGMP signaling, leading to higher activation of hypocrellin biosynthetic genes. Similar cooperative interactions between NO donors and other elicitors have been observed in several fungal and plant systems. For instance, in *S. bambusicola*, co-treatment with lanthanum ions and light–dark transition amplified hypocrellin A yields through ROS and NO co-signaling (Lu et al. 2019; Sun et al. 2018). Likewise, in plant cell cultures, simultaneous application of polyamines and NO donors synergistically enhanced alkaloid or phenolic production by coordinating ROS and cGMP-dependent transcriptional activation (Recalde et al. 2021). These findings support the view that controlled NO availability is a central determinant of elicitation efficiency in both fungal and plant secondary metabolism.

Compared with single-elicitor treatments, the combined Spd-SNP induction offers several advantages for biotechnological application. First, it enables a more stable and predictable intracellular NO level, avoiding the transient or excessive NO bursts that could otherwise induce oxidative damage or metabolic imbalance. Second, the co-elicitation simultaneously stimulates both primary carbon flux (via glycolysis and TCA cycle enhancement) and the transcription of secondary metabolic gene clusters, ensuring efficient precursor utilization for HA biosynthesis. Third, the dual elicitor system minimizes metabolic trade-offs between fungal growth and metabolite production, as demonstrated by the unaltered biomass and the selectively enhanced HA synthesis in *Shiraia sp.* S9. These advantages make Spd-SNP co-elicitation particularly attractive for process intensification in mycelium-based HA fermentation. From a bioprocessing perspective, integrating this dual-elicitation approach into bioreactor systems provides a promising route for scalable and sustainable production of hypocrellins. In submerged fermentation, NO donors can be precisely dosed through controlled feeding, while Spd can serve as a safe metabolic enhancer compatible with industrial media formulations. Such a strategy could overcome the low-yield limitations that have long hindered *Shiraia* biotechnological utilization (Li et al. 2025). Moreover, combining Spd-SNP elicitation with other process controls—such as oxygen supply modulation, ultrasound stimulation, or co-culture with beneficial bacteria—may further boost production efficiency through multi-signal integration. Therefore, the Spd-SNP co-elicitation unveiled in this study not only broadens the understanding of polyamine-NO signaling in fungi but also establishes a feasible and scalable elicitation paradigm for industrial hypocrellin production.

## Conclusions

Collectively, the present study establishes a comprehensive signaling framework in which Spd acts as a novel endogenous elicitor that enhances HA biosynthesis in *Shiraia sp.* S9 through a NO-dependent pathway. The results demonstrate that Spd activates dual NO-generating systems involving NOS and NR, leading to the accumulation of intracellular NO and subsequent stimulation of the downstream sGC- cGMP signaling cascade. This cascade reprograms the fungal transcriptome, promoting central carbon metabolism and the coordinated upregulation of key genes within the hypocrellin biosynthetic gene cluster, including *PKS*, *Mono*, *Omef*, and *ZFTF*. The integration of physiological, biochemical, and transcriptomic data supports a sequential regulatory model: Spd → NO → sGC/cGMP → transcriptional activation → HA biosynthesis, which expands the current understanding of perylenequinone regulation in *Shiraia*.

From a broader biological perspective, this work uncovers an evolutionarily conserved signaling logic between plants and fungi, in which polyamines and NO act as interlinked messengers to coordinate stress responses and secondary metabolism. Beyond the fundamental implications, this discovery provides a practical foundation for industrial application. The dual-elicitation strategy based on Spd and NO donors such as SNP offers a controllable, non-toxic, and metabolically compatible means to enhance hypocrellin yields in mycelium cultures. By integrating this regulatory insight with bioprocess optimization in bioreactors-combining precise elicitor timing, nutrient regulation, and oxygenation control-it is feasible to develop a scalable platform for the sustainable production of photoactive perylenequinones. In summary, this research thus introduces polyamines as a new class of metabolic elicitors in fungi and unveils the Spd-NO-cGMP cascade as a pivotal signaling axis for fungal secondary metabolism, paving the way for scalable production of photodynamically active perylenequinones for pharmaceutical and agricultural applications.

## Supplementary Information

Below is the link to the electronic supplementary material.

Supplementary Material 1

## Data Availability

Data will be made available on request.
