# Supplementary material for: Spermidine stimulates hypocrellin A biosynthesis through nitric oxide signaling in Shiraia sp. S9
Source: Bioresour Bioprocess. 2026 Apr 11;13(1):52. doi: 10.1186/s40643-026-01051-2 (PMC13070100; doi:10.1186/s40643-026-01051-2)
Supplement: Supplementary file 1 — Supplementary Material 1 [file 40643_2026_1051_MOESM1_ESM.pdf]

Supplementary materials

Spermidine stimulates hypocrellin A biosynthesis through nitric oxide signaling in *Shiraia* sp. S9

Li Ping Zheng, Rui Peng Cong, Xin Ping Li, Jian Qin Zhou, Jian Wen Wang

✉ Jian Wen Wang jwwang@suda.edu.cn

Table S1. Primers and relevant information of reference and target genes. F: forward primer, R: reverse primer.

Table S2. Effect of polyamine on perylenequinone production in PDA plates of *Shiraia* sp. S9.

Table S3. Illumina RNA-Seq reads and *de novo* assembly statistics results of *Shiraia* sp. S9 under Spd treatment.

Table S4. Examples of DEGs involved in the HA biosynthesis, carbon metabolism and polyamine metabolism of *Shiraia* sp. S9 under Spd, and Spd + cPTIO treatment.

Figure S1. The length distribution of unigenes obtained from Illumina RNA-Seq of *Shiraia* sp. S9 under Spd treatment. cPTIO (100 μM) was added 30 min prior to Spd treatment (1.0 mM) on day 4 of mycelium culture. The culture was maintained at 28°C and 150 rpm for 7 days.

Figure S2. Volcano plot of differentially expressed genes (DEGs) between samples. (**A**) Spd treatment vs. control. (**B**) Spd + cPTIO treatment vs. control. (**C**) Spd + cPTIO treatment vs. Spd. Analysis was carried out from the $\log_2$ normalized transcriptomic data, using a fold-change threshold of 1.0 and $p \leq 0.05$. Spd + cPTIO denotes samples treated with 100 μM cPTIO and 1.0 mM Spd.

Figure S3. Effects of different concentrations of SNP combined with the spermidine (Spd) treatment on biomass (**A**), HA content in mycelia (**B**), the released HA in cultural broth (**C**) and total HA production (**D**) of *Shiraia* sp. SNP (1-20 μM) was added 30 min prior to Spd treatment (1.0 mM) on day 4 of culture. The culture was incubated at 28°C and 150 rpm for 8 days. Values are mean ± SD from three independent experiments ($^{**}p < 0.01$ versus control, $^{\#}p < 0.05$ and $^{\#\#}p < 0.01$ versus Spd treatment).

Figure S4. Effects of addition time of SNP combined with the spermidine (Spd) treatment on biomass (**A**), HA content in mycelia (**B**), the released HA in cultural broth (**C**) and total HA production (**D**) of *Shiraia* sp. S9. SNP (5.0 μM) was added on day 3-7, and Spd (1.0 mM) was added on day 4 of culture. The culture was incubated at 28°C and 150 rpm for 8 days. Values are mean ± SD from three independent experiments ($^{**}p < 0.01$ versus control, $^{\#\#}p < 0.01$ versus Spd treatment).

Table S1 Primers and relevant information of reference and target genes. F: forward primer, R: reverse primer.

Genes symbol	Gene name	Sequence
18S	Reference gene	F: GAAAGTTAGGGGATCGAAGA<br>R: TAGTCGGCATAGTTTACGGT
MCO	Multicopper oxidase	F: TATGGCGCTACGAGTGGAC<br>R: ACTCCCTGGCCGATAACGTA
ZFTF	Zinc finger transcription factor	F: GAACACCGTCGCAAGATTCG<br>R: TCATTGGCATCGCTTGGAGT
Mono	Salicylate 1-monooxygenase	F: TCTCGGGGAATTATGGCACG<br>R: ACAACCGTTCTCGCATCAGT
FAD	FAD/FMN-containing dehydrogenase	F: TGTGACCGCCATCACCTTAC<br>R: TTGTCGTATGGGTGGGAAGC
Omef	O-methyltransferase	F: GAACTACCTGAAGGCACGCT<br>R: GCTCGGAAGGATACTCGCTC
PKS	Polyketide synthase	F: TGCTGAGGTAGCAGTCAAGC<br>R: TTATGCTACGGTCGTCGCTC
MFS	Major facilitator superfamily	F: TCCCGTAGCCTTGCTTTCTG<br>R: CCGGCTTCTTCTTGACGCTA
ABC	ATP-binding cassette	F: GACTTGAGCCTATCCGCCTC<br>R: AGAGTCGCCTCTGTGATCCT
NOS	Nitric oxide synthase	F: CCACTTGCCTAAGGACTC<br>R: CGCTATCGGGACTATGAC
NR	Nitrate reductase	F: AGGTATTGAGCGCCGACAAA<br>R: TGGACTTGCCACCAAACCTT

Table S2 Effect of polyamine on perylenequinone production in PDA plates of *Shiraia* sp. S9*.

PQ (mg/plate)	HA	HC	EA	EB	EC
Control	0.40±0.04	0.04±0.01	0.07±0.01	0.01±0.01	ND
Put	0.83±0.02**	0.10±0.01**	0.12±0.01*	0.01±0.01	0.01±0.01*
Spd	1.72±0.04**	0.24±0.02**	0.23±0.02**	0.03±0.01*	0.01±0.01*
Spm	1.29±0.11**	0.22±0.01**	0.22±0.02**	0.03±0.01*	0.02±0.01**

* The individual polyamine such as putrescine (Put), spermidine (Spd) or spermine (Spm) added respectively at 1.0 mM and the culture was maintained at 28 °C for 8 days. The culture without polyamine treatment was used as control. Values are mean ± SD from three independent experiments (* $p<0.05$ and ** $p<0.01$ versus control group). ND indicates no detection.

Table S3 Illumina RNA-Seq reads and de novo assembly statistics results of *Shiraia* sp. S9 under Spd treatment.

**Groups**	**Total clean reads**	**Base (bp)**	**GC (%)**	**Q20 (%)**	**Q30 (%)**
Control1	57535586	8473734337	50.84	99.07	96.38
Control2	69056508	10140746339	51.18	98.96	96.00
control3	70092798	10294619203	51.03	99.09	96.46
Spd1	53540332	7887339431	51.99	99.09	96.43
Spd2	62017592	9138896212	51.70	99.06	96.36
Spd3	68540790	10089206559	51.93	99.05	96.32
Spd+cPTIO1	63862774	9387220623	52.08	99.04	96.28
Spd+cPTIO2	56145044	8270630468	51.97	98.99	96.13
Spd+cPTIO3	57905092	8521208120	51.39	99.04	96.30

Table S4 Examples of DEGs involved in the HA biosynthesis, carbon metabolism and polyamine metabolism of *Shiraia* sp. S9 under Spd, and Spd + cPTIO treatment.

Gene ID	Up/Down		Fold change		Description
**Hypocrellin biosynthesis and transport**					
	Spd	Spd+cPTIO	Spd	Spd+cPTIO	
TRINITY_DN3431_c0_g1	Up	Up	11.49	4.42	Putative polyketide synthase
TRINITY_DN13421_c0_g1	Up	Down	1.16	-2.90	Asperfuranone polyketide synthase afog
TRINITY_DN16335_c0_g1	Up	Up	7.95	2.02	Conidial yellow pigment biosynthesis polyketide synthase
TRINITY_DN28558_c0_g1	Up	Up	3.47	1.03	Asperfuranone polyketide synthase afog
TRINITY_DN15730_c0_g1	Up	Up	3.15	2.92	Non-reducing polyketide synthase ausa
TRINITY_DN13569_c0_g1	Up	Up	3.41	3.28	Glandicoline B O-methyltransferase roqn
TRINITY_DN14541_c0_g1	Up	Up	2.34	1.98	O-methyltransferase
TRINITY_DN18950_c0_g1	Up	Up	5.68	3.69	Dimethyl-sulfide monooxygenase
TRINITY_DN15905_c0_g1	Up	Up	4.81	2.93	FAD-binding monooxygenase ausc
TRINITY_DN16881_c0_g1	Up	Up	4.73	2.44	Flavin-containing monooxygenase
TRINITY_DN23121_c0_g1	Up	Up	2.26	1.06	Flavin-binding monooxygenase-like
TRINITY_DN4415_c0_g1	Up	Down	2.55	-5.44	Monooxygenase activity
TRINITY_DN14191_c0_g1	Up	Down	1.13	-4.83	Monooxygenase activity
TRINITY_DN18528_c0_g1	Up	Up	2.29	1.96	Multicopper oxidase
TRINITY_DN18687_c0_g4	Up	Up	5.75	1.66	Zinc finger transcription factor ace1
TRINITY_DN30003_c0_g1	Up	Up	5.18	6.83	Isotrichodermin C-15 hydroxylase
TRINITY_DN20192_c0_g1	Up	Up	6.40	1.78	Major facilitator superfamily domain-containing protein
TRINITY_DN22261_c0_g1	Up	Up	4.44	1.03	MFS general substrate transporter
TRINITY_DN18774_c2_g6	Up	Down	2.28	-1.49	Uncharacterized MFS-type transporter C16A3.17c
TRINITY_DN14977_c0_g1	Up	Up	3.36	2.39	FAD-binding domain-containing protein
TRINITY_DN3307_c0_g1	Up	Up	2.36	1.57	FAD dependent oxidoreductase-like protein
TRINITY_DN13268_c0_g1	Up	Up	2.03	1.00	Uncharacterized FAD-linked oxidoreductase ARB_02478
TRINITY_DN29979_c0_g1	Up	Up	2.01	1.49	Fungal trichothecene efflux pump (TRI12)
TRINITY_DN16243_c0_g2	Up	Up	4.15	3.34	ABC transporter
TRINITY_DN20260_c0_g1	Up	Up	2.73	1.06	ABC transporter
TRINITY_DN12793_c0_g1	Down	Down	-1.04	-2.52	Dynamin family protein
**Glycolytic pathway (EMP)**					
TRINITY_DN14363_c0_g1	Up	Down	2.60	-4.77	Fructose-1,6-biphosphate aldolase (ALD)
TRINITY_DN2339_c0_g1	Up	Down	2.66	-5.54	Glyceraldehyde 3-phosphate dehydrogenase (GAPDH)
TRINITY_DN3463_c0_g1	Down	Down	-3.04	-2.74	Phosphoglycerate kinase (PGK)
TRINITY_DN29954_c0_g1	Up	Up	2.64	2.15	Phosphoglycerate mutase (PGAM)
TRINITY_DN16773_c0_g2	Down	Up	-2.74	1.94	Enolase (ENO)
TRINITY_DN18781_c0_g4	Up	Up	3.39	1.56	Pyruvate kinase (PK)
TRINITY_DN19181_c0_g3	Up	Up	2.13	2.22	Alcohol dehydrogenase (ADH)
TRINITY_DN2275_c0_g1	Up	Down	3.41	-1.10	Lactate dehydrogenase (LDH)
TRINITY_DN19107_c3_g3	Up	Up	2.05	1.37	Pyruvate decarboxylase (PDC)
TRINITY_DN23666_c0_g1	Up	Down	1.12	-3.14	Acetyl-CoA synthetase-like protein
**Tricarboxylic acid cycle (TCA)**					
TRINITY_DN24250_c0_g1	Up	Down	3.23	-1.32	2-Oxoglutarate dehydrogenase (OGDC)
TRINITY_DN19976_c0_g1	Up	Up	14.11	1.00	Succinate dehydrogenase (SDH)
TRINITY_DN26730_c0_g1	Up	Down	1.31	-2.51	Dihydrolipoyl dehydrogenase (DLD)
**Others**					
TRINITY_DN25607_c0_g1	Up	Up	4.62	1.85	Nitrate reductase (NR)
TRINITY_DN19087_c0_g1	Up	Up	2.72	2.38	Catalase
TRINITY_DN17576_c0_g1	Up	Up	3.29	1.23	Glutathione peroxidase

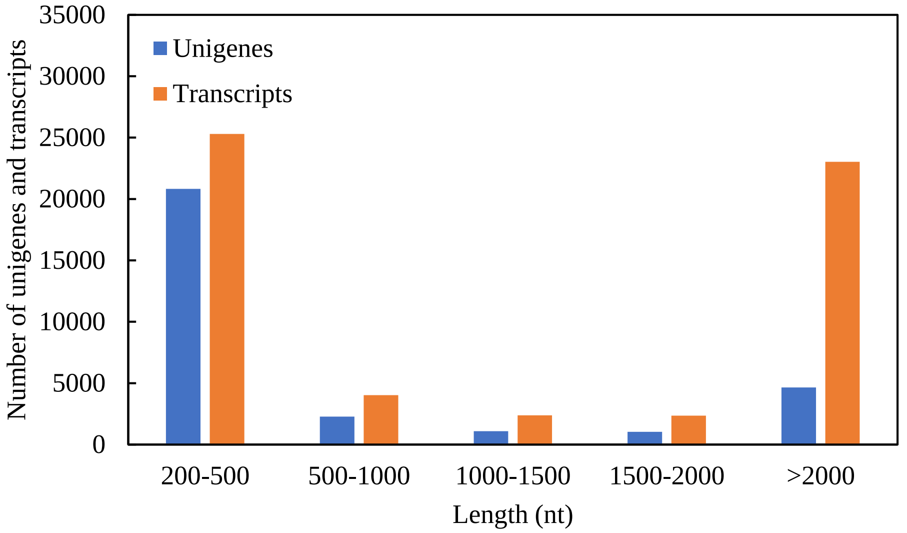

Fig. S1 The length distribution of unigenes obtained from Illumina RNA-Seq of *Shiraia* sp. S9 under Spd treatment. cPTIO (100 μM) was added 30 min prior to Spd treatment (1.0 mM) on day 4 of mycelium culture. The culture was maintained at 28℃ and 150 rpm for 7 days.

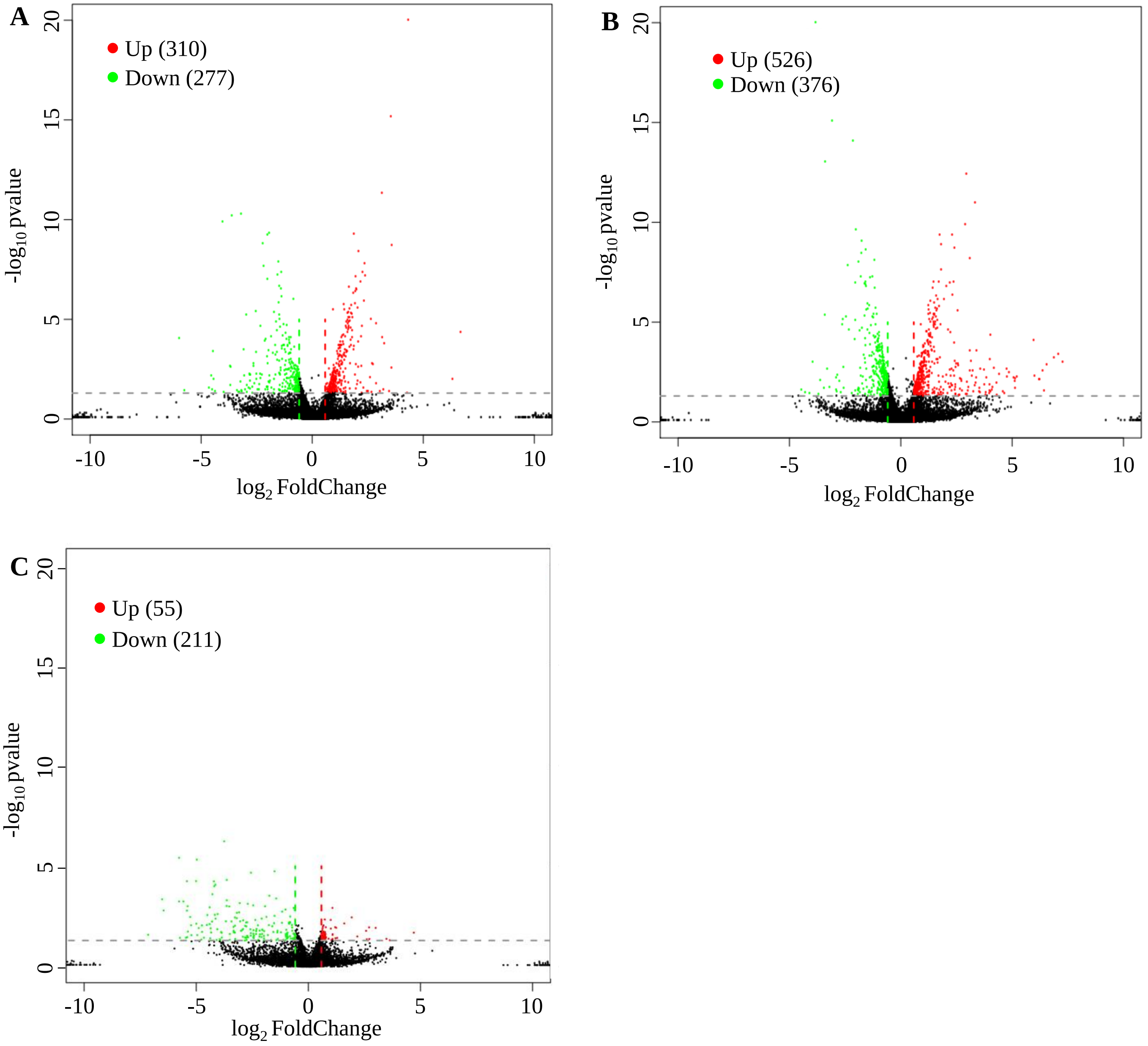

Fig. S2 Volcano plot of differentially expressed genes (DEGs) between samples. (**A**) Spd treatment *vs*. control. (**B**) Spd + cPTIO treatment *vs*. control. (**C**) Spd + cPTIO treatment *vs*. Spd. Analysis was carried out from the $\log_2$ normalized transcriptomic data, using a fold-change threshold of 1.0 and $p \leq 0.05$. Spd + cPTIO denotes samples treated with 100 μM cPTIO and 1.0 mM Spd.

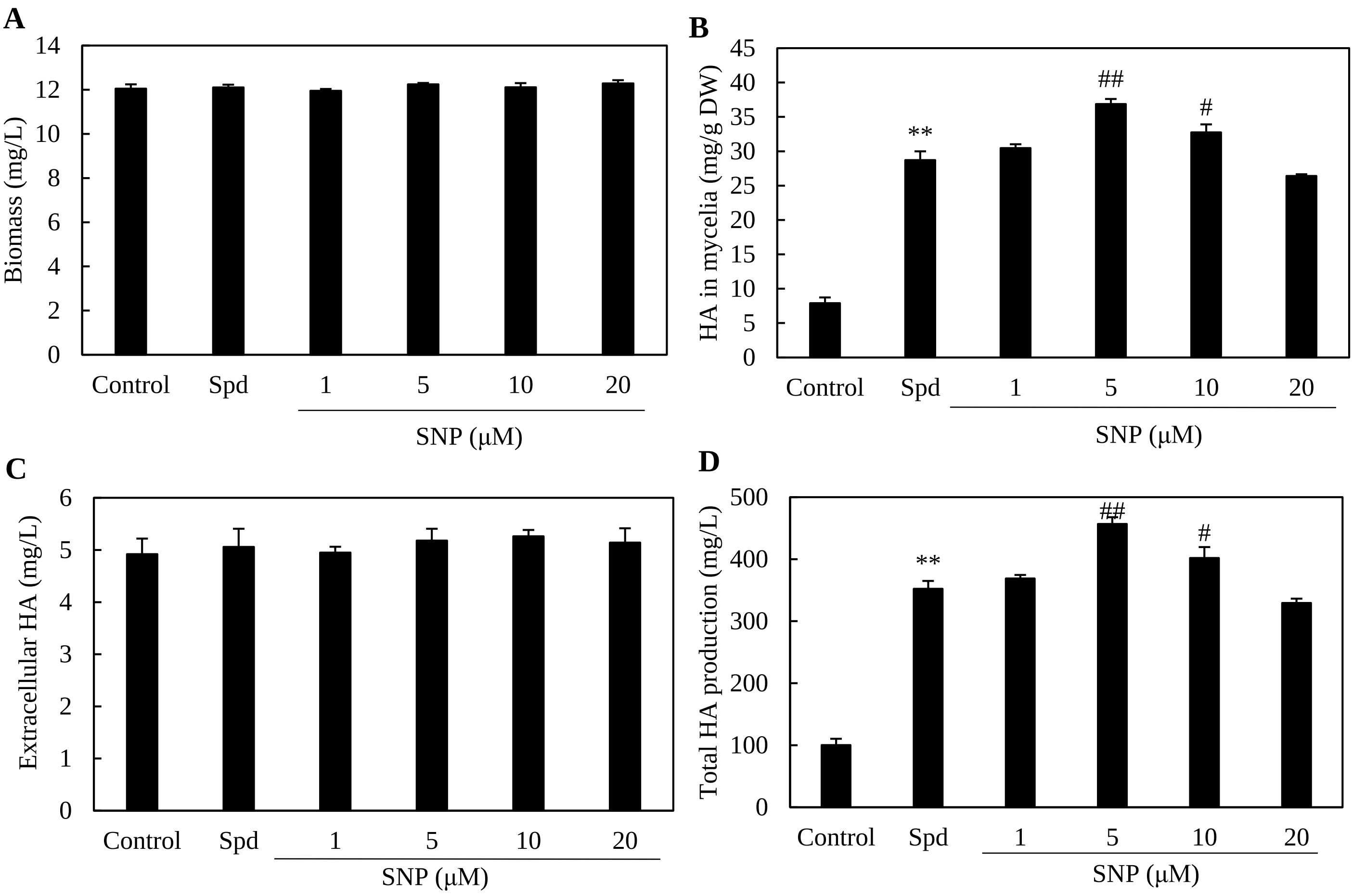

Fig. S3 Effects of different concentrations of SNP combined with the spermidine (Spd) treatment on biomass (**A**), HA content in mycelia (**B**), the released HA in cultural broth (**C**) and total HA production (**D**) of *Shiraia* sp. SNP (1-20 μM) was added 30 min prior to Spd treatment (1.0 mM) on day 4 of culture. The culture was incubated at 28°C and 150 rpm for 8 days. Values are mean ± SD from three independent experiments ($^{**}p < 0.01$ versus control, $^{\#}p < 0.05$ and $^{\#\#}p < 0.01$ versus Spd treatment).

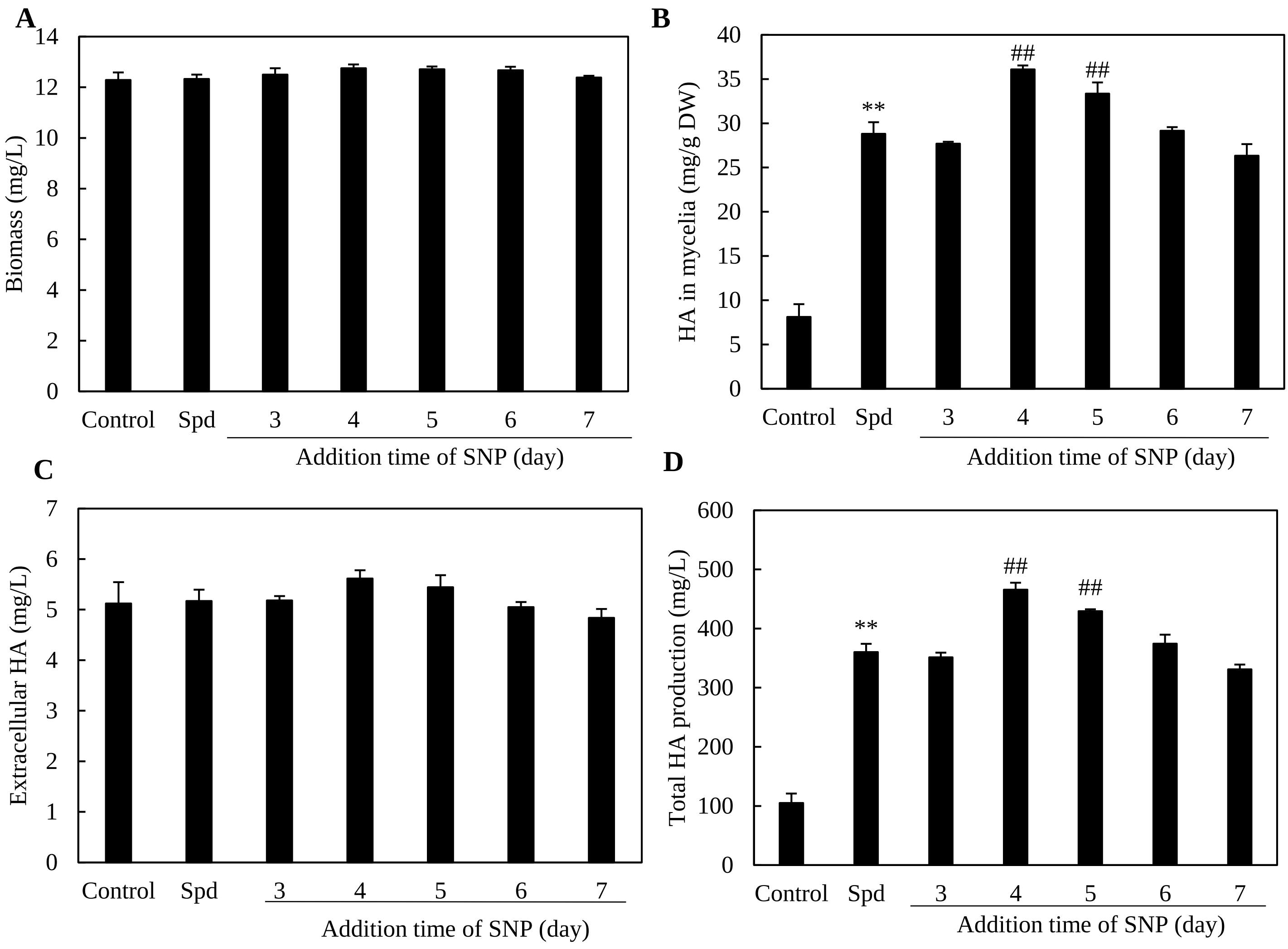

Fig. S4 Effects of addition time of SNP combined with the spermidine (Spd) treatment on biomass (**A**), HA content in mycelia (**B**), the released HA in cultural broth (**C**) and total HA production (**D**) of *Shiraia* sp. S9. SNP (5 μM) was added on day 3-7, and Spd (1.0 mM) was added on day 4 of culture. The culture was incubated at 28°C and 150 rpm for 8 days. Values are mean ± SD from three independent experiments ($^{**}p < 0.01$ versus control, $^{\#\#}p < 0.01$ versus Spd treatment).
